# *Rubus chingii* Hu. unripe fruits extract ameliorates carbon tetrachloride-induced liver fibrosis and improves the associated gut microbiota imbalance

**DOI:** 10.1186/s13020-022-00607-6

**Published:** 2022-05-12

**Authors:** Jianjun Wu, Dingqi Zhang, Bo Zhu, Siqi Wang, Yongbin Xu, Congcong Zhang, Hailing Yang, Shunchun Wang, Ping Liu, Luping Qin, Wei Liu

**Affiliations:** 1 College of Pharmaceutical Sciences, Zhejiang Chinese Medical University, Hangzhou, 310053 Zhejiang China; 2 Key Laboratory of Liver and Kidney Diseases (Ministry of Education), Institute of Liver Diseases, Shuguang Hospital, Shanghai University of Traditional Chinese Medicine, 528 Zhangheng Road, Shanghai, 201203 China; 3 The MOE Key Laboratory for Standardization of Chinese Medicines and The SATCM Key Laboratory for New Resources and Quality Evaluation of Chinese Medicines, Institute of Chinese Materia Medica, Shanghai University of Traditional Chinese Medicine, 1200 Cailun Rood, Shanghai, 201203 China

**Keywords:** *Rubus chingii* Hu., Liver fibrosis, Hepatic stellate cells, TGF-β/Smads, Gut microbiota

## Abstract

**Background:**

The unripe fruits of *Rubus chingii* Hu. (“*Fu-peng-zi*” in Chinese) is a well-known herbal tonic in traditional Chinese medicine (TCM) for tonifying liver and kidney. However, little is known regarding its therapeutic efficacy against liver fibrosis and the underlying mechanism.

**Methods:**

The current research aims to explore the potential of *Rubus chingii* Hu. unripe fruits extract (RF) in the treatment of liver fibrosis and explore the underlying mechanism. RF was administered (450 and 900 mg·kg^− 1^ of body weight per day) orally to male C57BL/6 mice with CCl_4_-induced liver fibrosis for 3 weeks. The histopathological changes and fibrosis stage in liver tissue were assessed using hematoxylin and eosin (H&E) and Sirius red staining. The distribution of α-SMA and Col1A1 in the liver was analyzed to determine the hepatic stellate cells (HSCs) activation using immunohistochemistry and immunofluorescent analysis. Various biochemical markers in serum (ALT, AST) and liver (Hyp, IL1-β, IL6, TNF-α and MCP-1) were observed to assess the liver’s injury, fibrosis, and inflammation. In liver tissue, fibrosis-associated proteins including α-SMA, TGF-β1, Smad2/3, p-Smad2/3, and Smad4 were detected through a Western blot assay. Pyrosequencing-based analysis of bacterial 16 S ribosomal RNA from variable regions V3–V4 of fecal samples characterized the gut microbiota. Spearman’s rank correlation analysis was performed for the association between altered bacterial genera by RF and pharmacodynamics parameters.

**Results:**

Three weeks of RF treatment can significantly lower liver inflammatory levels, pathological abnormalities, and collagen fibrous deposition in mice with CCl_4_-induced liver fibrosis. The expressions of α-SMA and Col1A1 were lowered by RF, while the expression levels of TGF-β/Smads signaling pathway-related proteins, including TGF-β1, p-Smad2/3, and Smad4, were dramatically decreased by RF. The RF treatment significantly increased or reduced 18 different bacterial species, restoring the CCl_4_-induced gut microbiota imbalance to the normal group’s levels. According to correlation analysis, the bacterial genera Bifidobacterium and Turicibacter were the most significant in restoring CCl_4_-induced liver fibrosis.

**Conclusions:**

RF can reduce liver damage and delay the onset of liver fibrosis through modulating TGF-β/Smads signaling pathway. Furthermore, RF’s anti-liver fibrosis effect was related to balancing the gut microbial community, partly attained by increasing Bifidobacterium and Turicibacter in liver fibrosis.

**Supplementary Information:**

The online version contains supplementary material available at 10.1186/s13020-022-00607-6.

## Introduction

Liver fibrosis is a pathological process involving the abnormal development of fibrous connective tissue in the liver when liver cells are necrotic and is driven by inflammation. As a frequent pathological basis of numerous hepatic diseases, liver fibrosis may culminate in end-stage liver disease or cirrhosis, contributing to irregular liver function and, finally, liver failure [1]. This tendency affects 1–2% of the global population, resulting in over 1 million fatalities each year [2, 3]. Effective prevention or alleviation of liver fibrosis is a real challenge in treating chronic liver illnesses. However, there is currently no effective medical intervention for the prevention and/or treatment of hepatic fibrosis with a lower risk of side effects. As a result, a safe and effective method for preventing or treating liver fibrosis is urgently needed.

Liver fibrosis is defined as a wound-healing reaction characterized by a large amount of extracellular matrix (ECM) formation and deposition, such as collagen, fibronectin, and proteoglycan [4, 5]. Hepatic stellate cells (HSCs), the primary mesenchymal cells in the liver and the starting point of fibrogenic cells, have a critical role in hepatic fibrosis pathogenesis [6]. When the liver is damaged, many signaling pathways concentrate on HSCs, promoting transdifferentiation to an active myofibroblast and phenotypic alterations that lead to ECM deposition. HSCs that have been activated are the focus of antifibrotic therapy. It might be employed to treat liver fibrosis in a clinical setting if HSCs activation could be decreased, or perhaps the active status of HSCs could be driven back to a static condition [1, 7, 8].

Recent research has proposed a crucial role of gut microbiota in the pathogenesis and progression of various liver disorders, including liver fibrosis. Mazagova et al. reported the effect of gut microbiota on the development of liver fibrosis after thioacetamide or CCl_4_-induced toxic liver damage. Germ-free mice developed higher liver fibrosis than normal mice. Hepatocytes from germ-free animals showed increased toxin-induced oxidative stress and cell death and increased HSCs activation [9]. Boursier et al. observed that the structure and function of the gut microbiota of patients with various degrees of liver fibrosis change significantly [10]. Patients with intermediate or high-grade fibrosis had higher abundance of Bacteroides and Ruminococcus, and lower abundances of Prevotell, compared to patients with no fibrosis or low degree fibrosis. Janssen et al. found that guar gum feeding affected the gut microbiota composition and was associated with increased hepatic inflammation and fibrosis in non-alcoholic fatty liver microscopic models [11]. Unlike guar gum, persistent antibiotic therapy significantly suppressed gut bacteria and reduced hepatic inflammation and fibrosis in non-alcoholic fatty liver mice. These findings revealed a causal relationship between gut microbiota and liver fibrosis, as well as a new approach for preventing and treating liver fibrosis.

*Rubus chingii* Hu. (“*Fu-pen-zi*” in Chinese) belonging to the Rosaceae family and is a perennial crop that is extensively distributed in China [12]. The unripe fruits of *R. chingii* (also known as Rubi Fructus in Chinese Pharmacopoeia) are a well-known herbal tonic which has been widely used in traditional Chinese medicine (TCM) for over 1500 years and are still widely used today. As recorded in many important Chinese medical monographs, such as “*Kai Bao Ben Cao*”, “B*en Cao Fa Ming*”, “*Ben Cao Qiu Yuan*”, “*Ben Cao Bei Yao*”, “*Ben Cao Cong Xin*”, and Chinese Pharmacopoeia (2020 edition), Rubi Fructus was traditionally used to nourish the liver and tonify the kidney. The extract of Rubi Fructus has been demonstrated in recent pharmacological studies to have hepatoprotective effects [13], free radical scavenging [14–16], antioxidant [17–19], anti-inflammation [20–22], hypoglycemic effects [13, 23], and other significant biological activities [24–26]. However, no study on the effects of Rubi Fructus extract on liver fibrosis has yet been reported.

Therefore, this study investigates the effect of Rubi Fructus extract on biochemical abnormalities and gut microbiota in mice with CCl_4_-induced liver fibrosis. And to examine the potential mechanisms for reducing liver fibrosis by inhibiting HSCs activation, pharmacodynamic parameters, and gut microbiota correlations, and to identify different gut microbiota related to Rubi Fructus’ anti-liver fibrosis effect.

## Materials and methods

### Chemicals and reagents

Aspertate aminotransferase (AST) test kit, alanine aminotransferase (ALT) test kit were purchased from Nanjing Jiancheng Bioengineering Institute (Nanjing, China), CCl_4_ (Lot#P1493460) acquired from Shanghai Titan Scientific Co., Ltd. (Shanghai, China). Hematoxylin and Eosin (H&E) staining kit, and hydroxyproline (Hyp) assay kit. Interleukin-1β (IL-1β), interleukin-6 (IL-6), tumor necrosis factor-α (TNF-α), and monocyte chemoattractant protein-1 (MCP-1) ELISA kits were purchased from Enzyme-linked Biotechnology Co., Ltd. (Shanghai, China). The immunohistochemistry kit (Cat#GK500705) was acquired from Gene Tech Company Limited (Shanghai, China). Antibodies against Smad2/3 (Cat#8685S), p-Smad2/3 (Cat#8828S), and GAPDH (Cat#51332S) were obtained from Cell Signaling Technology (Danvers, MA, USA). The anti-TGF-β1 (Cat#ab92486), α-smooth muscle actin (α-SMA, Cat#ab5694) and Smad4 (Cat#ab40759) were obtained from Abcam, Inc. (Cambridge, UK). Type I collagen (Col1A1, Cat#A16891) was acquired from ABclonal Biotechnology Co., Ltd. (Wuhan, China). PVDF membrane was obtained from Millipore Corp. (Bedford, MA, USA). Shanghai Baishi Kai Chemical Technology Co., Ltd. (Shanghai, China) supplied Sorafenib Tosylate (purity > 99%). Gallic acid, isoquercitrin, ellagic acid, hyperoside, rutin, kaempferol-3-rutinoside, quercetin, luteolin, and tiliroside were acquired as reference standards from Chengdu Biopurify Phytochemicals Ltd. (Chengdu, China), and their purities were greater than 98%.

### Collection and preparation of plant material

The unripe fruits of *R. chingii* was collected in Chun’an county, Zhejiang, China and authenticated by professor Luping Qin, College of Pharmaceutical Sciences, Zhejiang Chinese Medical University. Fresh herbs were dried in shade for a week. Dried *R. chingii* unripe fruits (500 g) was extracted with 20 times the volume of 50% ethanol (*v/v*) in reflux conditions for 3 times, each for 2 h. The extract solutions were mixed, filtered, and concentrated under reduced pressure at 45 ℃. Finally, the powder of *R. chingii* unripe fruit extract (RF, 94.60 g) was obtained through the freeze-drying method.

RF (0.5 g) was weighed and dissolved in 50 mL deionized water by ultrasonic (250 W, 50 Hz) for 1 h. Then, the sample solution was centrifuged at 10,000×*g* for 15 min. The supernatant solution was filtered through a 0.22 μm membrane, and the chemical constituents in the extract of RF were identified by ultra-high-performance liquid chromatography-Q exactive hybrid quadrupole orbitrap high-resolution accurate mass spectrometric (UHPLC-Q-Exactive Orbitrap HRMS). And then the contents of mainly constituents gallic acid, isoquercitrin, ellagic acid, hyperoside, rutin, kaempferol-3-rutinoside, quercetin, luteolin and tiliroside were determined by UHPLC-Q-Exactive Orbitrap HRMS.

### Chromatography conditions for UHPLC-Q-Exactive Orbitrap HRMS

The UHPLC-Q-Exactive Orbitrap system was used for chromatographic separation (Thermo Fisher Scientific Inc., Grand Island, NY, USA). The UHPLC system is comprised of a Chromeleon 7.2 Software-controlled Thermo Scientific Dionex Ultimate™ 3000 Series RS pump, TCC-3000 RS column compartments, and WPS-3000 autosampler from Thermo Scientific Dionex Ultimate™ 3000 Series. The cooling autosampler was kept at 4 °C and kept out of direct sunlight, while the column heater was kept at 40 °C. The column used was a Waters ACQUITY UPLC BEH C_18_ column (2.1 × 100 mm, 1.7 μm). The mobile phase was A (methanol) and B (0.1% formic acid) at a flow rate of 0.3 mL·min^− 1^ and eluted with gradient elution: 0–4 min (4% A), 4–10 min (4 -12% A), 10–28 min (12 -64% A), 28–30 min (95% A), 30–32 min (4% A). The injection was 2 µL in volume.

Xcalibur 4.1 software was used to control the mass spectrometer Q-Exactive Orbitrap system, which was coupled to the UHPLC system through heated electrospray ionization. A negative ionization mode was used to operate and optimize the electrospray ionization source. The optimized parameters of mass spectrometry were: sheath gas (N_2_) flow rate: 45 arbitrary units; capillary temperature: 325 °C; sweep gas flow rate: 0 arbitrary units; auxiliary gas (N_2_) flow rate: 8 arbitrary units; S-lens RF level: 50 V; spray voltage: 2.8 kV; scan mode: Full MS/SIM mode (resolution 70,000 FWHM); auxiliary gas heater temperature, 300 °C; scan range: 80-1200 m/z; automatic gain control (AGC) target: 1.0 e^6^.

### Experimental animals and drug administration

The procedure of animal experiment was approved (PZSHUTCM191101010; Approval date: 01 November 2019) and supervised the Shanghai University of Traditional Chinese Medicine’s Animal Ethics Committee, which was carried out following the People’s Republic of China’s State Committee of Science and Technology guidelines (14 November 1988). Eight-week-old C57BL/6J male mice (20 ± 2 g) were purchased from Beijing Vital River Laboratory Animal Technology Co., Ltd. (Beijing, China) and fed in the Shanghai University of Traditional Chinese Medicine’s Experimental Animal Center. Mice were accustomed to the surroundings for one week before the experiment, with unrestricted access to water and food. All of the animals were separated into two groups: the control (n = 7) and the hepatic fibrosis model (n = 28). Intraperitoneal injection of 15% CCl_4_ diluted in olive oil at doses of 2 mL/kg body weight thrice weekly for 6 weeks developed a liver fibrosis model in accordance with a previous report [27]. Meanwhile, normal control mice were received 2 mL/kg body weight intraperitoneal injection of olive oil. CCl_4_-induced mice were randomly separated into 4 groups after 3 weeks of CCl_4_ treatment: CCl_4_ fibrosis model group (n = 7), low-dose RF group (RFL, n = 7, 450 mg/kg/d, p.o, a simple conversion based on body weight was used to derive the animal dose from the human daily dose), high-dose RF group (RFH, n = 7, 900 mg/kg/d, p.o), and sorafenib positive control group (Sora, n = 7, 10 mg/kg/d, p.o). Mice enrolled in the model, and the control groups were given the same quantity of 0.3% CMC-Na. After the 6th week, all animals were euthanized under anesthesia, and the liver tissues and serum specimens were taken for further study.

### Serum biochemistry and hepatic Hyp content analysis

The activities of ALT and AST in the serum, and the content of Hyp in the liver tissues, were estimated following the manufacturers’ instructions for the respective analytical kits. The amount of Hyp was measured in µg/g of wet weight.

### Histopathological assay

Liver tissues were fixed in 10% formalin, followed by dehydration in a LEICA ASP300S automated vacuum tissue processor (LEICA Microsystems, Wetzlar, Germany). Following the manufacturer’s instructions, they were then paraffin-embedded and sliced at 4 μm thickness for H&E and Sirius red staining. Histopathological changes and fibrosis stage were assessed using H&E and Sirius red staining. Sirius red staining was also quantified using Leica LAS Image Analysis.

### Immunohistochemistry and immunofluorescent analysis

The tissue sections (4 μm) were de-waxed with xylene and re-hydrated with gradient ethanol. The sections were heated in citrate buffer (pH 6.0) at 100 °C for 8 min and then at 60 °C for 25 min for antigen retrieval. Endogenous peroxidase was blocked for 10 min with 3% H_2_O_2_ methanol solution, and nonspecific antibody binding was blocked for 30 min using 10% goat non-immune serum, before the sections were incubated using primary antibodies against α-SMA (1:1000) and Col1A1 (1:500) at 4 °C overnight, respectively. The specimens were then lightly counterstained with hematoxylin and treated with secondary antibody for 30 min before DAB staining. Finally, these specimens were scanned, and representative images were recorded using a LEICA SCN400F scanner (LEICA Microsystems, Wetzlar, Germany).

Liver specimens were embedded in an optimal cutting temperature compound, sliced at an 8-millimeter thickness, and preserved in cold acetone for 10 min, as well as blocked using 10% goat non-immune serum for 30 min. At 4 °C overnight, the sections were treated with primary antibodies against α-SMA (1:1000) and Col1A1 (1:500), respectively. The specimens were then stained with DAPI and fluorescent secondary antibodies (1:3000). The Olympus Fluoview 500 confocal microscope (Olympus, Malvern, USA) was used to scan these specimens, producing representative images.

### ELISA assay

Liver tissue (100 mg) was homogenized in 1 mL PBS and was centrifuged at 3000 rpm for 20 min, respectively. The levels of IL1-β, IL6, TNF-α, and MCP-1 from the supernatant of centrifuged homogenates were measured using ELISA kits following the protocol defined by the manufacturer.

### Western blot assay

Liver tissues were lysed and homogenized in 800 µL RIPA lysis buffer (including phosphatase and protease inhibitors), followed by centrifugation at 12,000 rpm for 20 min at 4 °C. BCA assay kit was employed for the determination of the protein content. The protein samples (30 µg) were then prepared using SDS-PAGE protein loading buffer (5×), water, and 10% SDS-polyacrylamide gel electrophoresis to separate them. The separated proteins were blotted onto PVDF membranes, which were then soaked in quick sealing fluid for 30 min before being incubated with primary antibodies such as α-SMA (1:1000), TGF-β1 (1:1000), Smad2/3 (1:1000), p-Smad2/3 (1:1000), and Smad4 (1:1000) overnight at 4 °C (1:1000). The blots were later incubated at room temperature for an hour with a secondary antibody (1:5000) before being scanned with an Odyssey infrared imaging equipment (LI-COR, Biosciences, UK). Image J was used to quantify the bands (National Institute of Health, Bethesda, MD, USA).

### Gut microbiota analysis

QIAamp DNA Stool Mini Kit (Qiagen, Hilden, Germany) was employed for the extraction of bacterial genomic DNA from frozen stool specimens in accordance with the protocol detailed by the manufacturer. Thermal cycling for the amplification of the 16S rRNA of the V3-V4 area (338F:5′- ACTCCTACGGGAGGCAGCAG-3′; 806R:5′- ACTCCTACGGGAGGCAGCAG-3′) was as follows: 3 min initial denaturation at 95 °C, 27 cycles (30 s at 95 °C and 30 s at 55 °C), 72 °C for 10 min. The PCR was done in triplicates. Purified template DNA (10 ng), 5 × FastPfu Buffer (4 µL), dNTPs (2 µL, 2.5 mM), forward and reverse primers (0.8 µL, 5µM), FastPfu Polymerase (0.4 µL), BSA (0.2 µL), and nuclease-free water (20 µL) made up the PCR reaction mixture.

Agarose gels (2%) were used to extract the amplicons which were purified using the AxyPrep DNA Gel Extraction Kit (Axygen Biosciences, Union City, CA, US) as per the manufacturer’s instructions. Quantification of the amplicons was then carried out using the QuantiFluorTM-ST as described in the manufacturer’s instructions (Promega, US). After demultiplexing the raw fastq files, we deposited them in the NCBI Sequence Read Archive (SRA) database. Apart from that, the Raw fastq files were filtered for quality using Quantitative Insights into Microbial Ecology (QIIME, version 1.17) according to the following criteria: (i) The quality score of < 20 over a 50 bp sliding window was employed for truncating the 300 bp reads at any individual site, and the truncated reads shorter than 50 bp were disregarded; (ii) a barcode with an exact match, a primer match with two nucleotide mismatch, and reads with ambiguous characters were discarded; (iii) Sequences having overlap greater than 10 bp were assembled as per their overlap sequence whereas the reads not qualifying the assembling criteria were discarded. The operational taxonomic units (OTUs) were clustered using UPARSE version 7.1 (https://drive5.com/uparse/) with a 97% similarity cutoff using the UPARSE software. The chimeric sequences had to be identified and removed, which was done with the help of UCHIME. Furthermore, we used the RDP Classifier (https://rdp.cme.msu.edu/) to analyze the taxonomy of each 16 S rRNA gene sequence against the silva (SSU115) 16 S rRNA database with a 70% confidence threshold.

### Data analysis

The mean, standard deviation (mean ± SD) was used to express the data in the text. One-way ANOVA was used to compare the groups in the data, followed by Fisher’s LSD post hoc test in GraphPad Prism version 6. *P* < 0.05 was deemed statistically significant. Prior to statistical analysis, we used a log10 transformation to normalize the copies of the 16 S rRNA gene. The QIIME program was used to calculate the β-diversity of unweighted UniFrac principal coordinate analysis (PCoA). Based on log10 LDA > 2.0, the linear discriminant analysis (LDA) effect size (LEFSe) technique was employed to investigate significant differences among a pair of groups of bacterial genera. Further, we employed spearman’s rank correlation analyses to investigate the relationship between gut microbiota and pharmacodynamics parameters.

## Results and discussions

### Preparation of RF

**Fig. 1:**
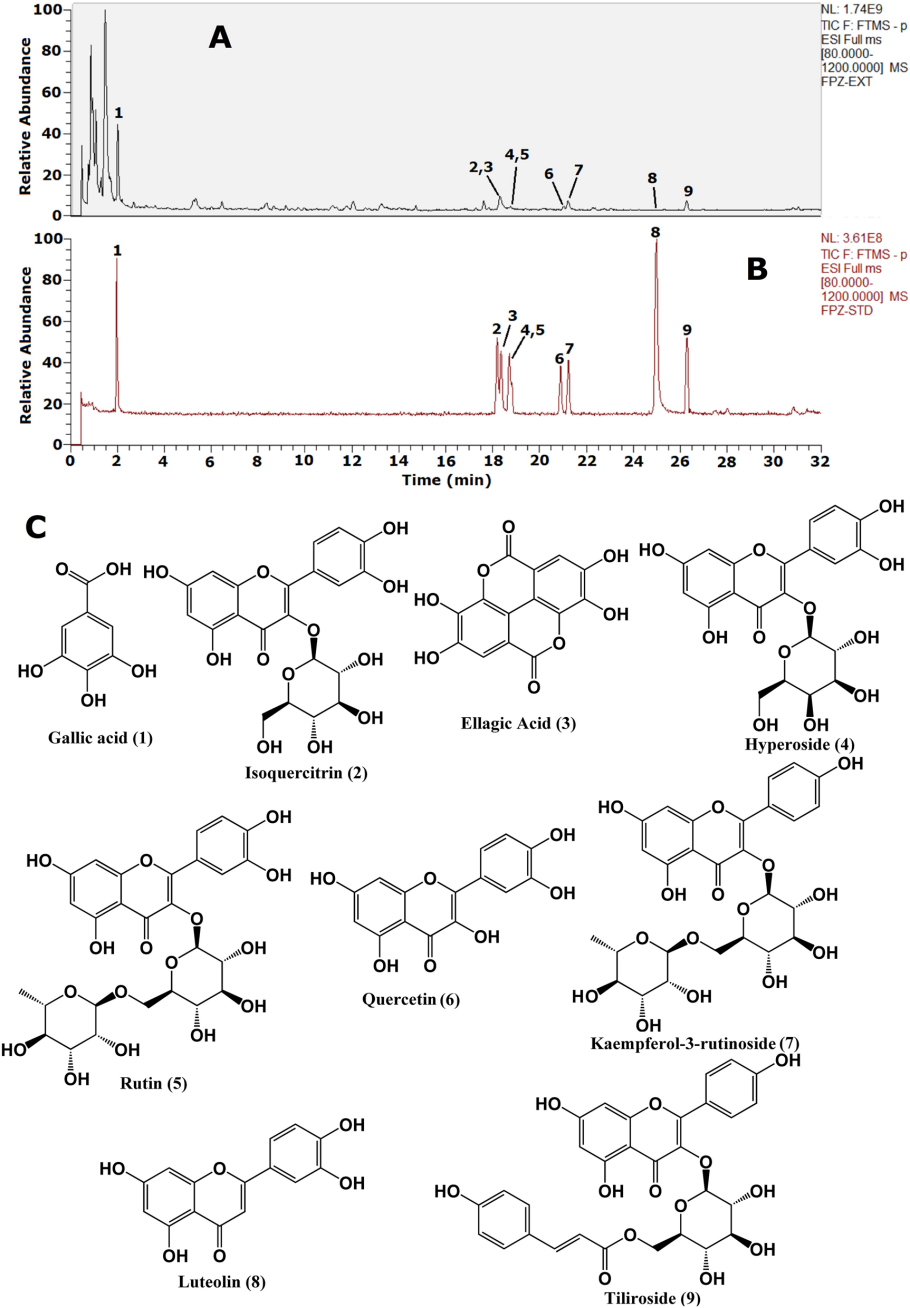
Structural elucidation of the extract of *Rubus chingii* Hu. unripe fruits (RF) using UHPLC-Q-Exactive Orbitrap HRMS. **A** the total ion chromatograms (TICs) of RF in negative mode; **B** TICs of mixture reference standards in negative mode; **C** the chemical structures of target reference standards

A total of 89 chemicals were quickly and accurately identified from RF (Additional file 1: Fig. S1), via comparison with the retention times and MS/MS spectra of the reference standards, reference literatures, Chemical Book and other databases. The detailed information of the identified chemical constituents in RF were listed Additional file 1: Table S2. And then under the optimal conditions described above, the total ion chromatograms (TICs) of RF and target reference standards in negative mode were shown in Fig. 1. The contents of targeted markers gallic acid, isoquercitrin, ellagic acid, hyperoside, rutin, quercetin, kaempferol-3-rutinoside, luteolin and tiliroside in RF were determined as 6.056 mg/g, 0.026 mg/g, 18.390 mg/g, 0.142 mg/g, 0.377 mg/g, 0.929 mg/g, 1.591 mg/g, 0.0006 mg/g and 1.381 mg/g, respectively.

### RF alleviates CCl_4_-induced liver damage and inflammatory injury in mice

CCl_4_ is a strong hepatotoxin that causes lipid peroxidation in hepatocytes, leading to activation of HSCs, disturbances in lipoproteins synthesis, damage to mitochondria, and disorders in lipid metabolism in hepatocytes [28]. Moreover, the prolonged stimulation of CCl_4_-induced the accumulation of Col1A1 in the liver leads to liver fibrosis. CCl_4_, as a liver injury and fibrosis promotor, is widely used for establishing animal liver fibrosis models [29–31]. We used a mouse liver fibrosis model developed by intraperitoneal injection of CCl_4_ and harvested tissues after the last RF treatment with doses of 450 and 900 mg·kg^− 1^ to explore the anti-fibrosis efficacy of RF.

**Fig. 2:**
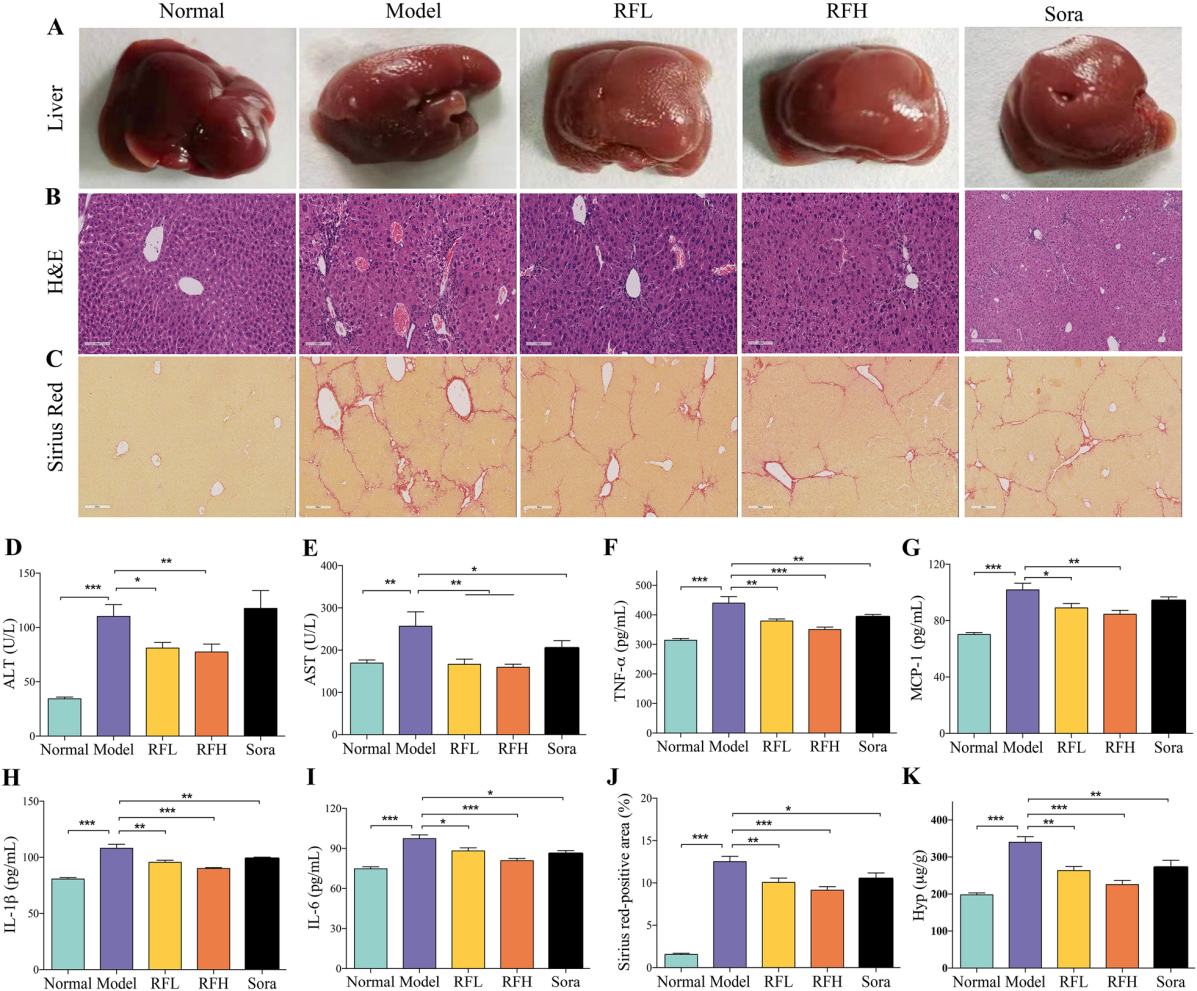
RF alleviates CCl_4_-induced liver damage, inflammatory injury and liver fibrosis in mice. **A** Representative photographs of livers in each group. **B** H&E (× 200) staining and **C** Sirius red (× 100) staining. Serum activities of **D** ALT and **E** AST. Liver **F** TNF-α, **G** MCP-1, **H** IL-1β, and **I** IL-6 levels. **J** Sirius red-positive area (%). **K** Hyp content. The results are expressed as the mean ± SD. ^*^p < 0.05, ^**^p < 0.01, ^***^p < 0.001 versus the CCl_4_ group

Pathological changes in the liver were observed after the experiment (Fig. 2A). The liver of normal mice has a normal brilliant red appearance, with a smooth surface and soft and elastic texture, and no noticeable swelling. The liver was found to have a rough, elevated grain surface, was more fragmented, and noticeably swollen after CCl_4_ induction. The surface of the liver was smoother, the texture was softer, and there was no apparent swelling after RF therapy compared to the model group. H&E staining revealed abnormalities in liver histopathology in mice (Fig. 2B). The results revealed morphological abnormalities in the livers of CCl_4_-induced mice, including hepatic cord disorder, lobular structure disorder, hepatocyte swelling and degeneration, and a significant number of inflammatory cells infiltration in the portal zone. The dose-dependent decrease of CCl_4_-induced hepatic pathological abnormality was seen after 3 weeks of RF treatment.

Clinically, the levels of ALT and AST, two typical serum markers of liver injury, are often used to evaluate liver function indirectly. In comparison to the control group, serum ALT (*p* < 0.001, Fig. 2D) and AST (*p* < 0.01, Fig. 2E) levels were observed to increase in mice with CCl_4_-induced fibrosis, indicating that the hepatocytes are damaged. After RF treatment, both RFL and RFH can lead to a significant decrease in serum ALT (*p* < 0.05 for RFL; *p* < 0.01 for RFH) and AST (*p* < 0.01 for RFL; *p* < 0.01 for RFH) levels. These results demonstrate that RF can cause a significant improvement in liver injury.

Chronic inflammation has a significant role in the progress of hepatic fibrosis and is thought to be a key mediator. Overproduction of proinflammatory cytokines damages hepatocytes directly and promotes liver fibrosis with the gradual replacement of the scar tissue with the normal hepatocyte structure [32]. We examined the effectiveness of RF in inhibiting chronic inflammation by measuring the levels of TNF-α, MCP-1, IL-1β, and IL-6 in liver tissue by ELISA assay. The results indicated that the pro-inflammatory factors TNF-α (*p* < 0.001, Fig. 2F), MCP-1 (*p* < 0.001, Fig. 2G), IL-1β (*p* < 0.001, Fig. 2H), and IL-6 (*p* < 0.001, Fig. 2I) were expressed in significantly higher levels in the CCl_4_ model group (p < 0.001) than in the normal control group. RF-treated mice, on the other hand, had considerably lower levels of these inflammatory factors (*p* < 0.05, *p* < 0.01, or *p* < 0.001). These results suggest that RF caused a reduction in the inflammatory levels in CCl_4_-induced liver fibrosis mice.

### RF alleviates CCl_4_-induced liver fibrosis in mice

Collagen fibers in the liver continue to accumulate during the fibrosis process. Sirius red dye is an acidic solid anion dye that readily binds to the basic groups of collagen molecules. Collagen is stained red under a regular light microscope, while muscle fibers and cytoplasm are dyed yellow. Sirius red staining is frequently employed in liver fibrosis models for semi-quantitative measurement of collagen fibrous deposition. Red collagen fiber deposition was uncommon in the normal group, as illustrated in Fig. 2C. In the model group, a considerable number of red collagen fibers were deposited around the portal region and created a thick fibrous septum, some of which penetrated deep into the liver’s interior lobules, as compared to the normal group. After RF treatment, there was still more collagen fibrous deposition in the portal area in the RFL group, but fewer collagen fibers and fibrous septum than in the model group. In the RFH group, the collagen fibrous fibers were significantly reduced and confined to the portal area. Furthermore, sirius red-positive area (SR) was calculated to evaluate collagen deposition in hepatic fibrosis (Fig. 2J). When compared to the CCl_4_ treatment group, SR was considerably reduced after treatment with RF and Sorafenib (*p <* 0.01 for RFL; *p <* 0.001 for RFH; and *p <* 0.05 for Sorafenib). The results suggested that RF can alleviate the collagen fibrous deposition induced by CCl_4_ in the liver, thus improving liver fibrosis progression.

Collagen is continuously accumulated in liver fibrosis and is composed of 18 kinds of amino acids, among which Hyp is the prevalent amino acid in collagen. Consequently, the determination of Hyp content can reflect the level of liver collagen accumulation, which can then be used to determine the degree of liver fibrosis [33, 34]. Compared to the control group, the CCl_4_ group had significantly higher Hyp content (*p <* 0.001, Fig. 2K), indicating the excessive collagen deposition in the liver during fibrosis, which is consistent with previous findings. After RF and Sora treatment, Hyp content was distinctly reduced (*p <* 0.01 for RFL and Sora; *p <* 0.001 for RFH). As a result, the findings imply that RF is critical in preventing collagen deposition during fibrosis. These findings show that RF substantially reduces liver fibrosis in CCl_4_-induced mice.

### RF ameliorates the expressions of α-SMA and Col1A1 in the liver of mice with CCl_4_-induced liver fibrosis

In the case of liver fibrosis, HSCs are the predominant cell type that participates in excessive collagen formation. The activation of HSCs is a critical event in liver fibrosis and inhibiting it is crucial for fibrosis relief [35]. Activated HSCs can be transformed into myofibroblasts with the properties of promoting fiber proliferation, and then the expressions of α-SMA and Col1A1. The higher the activation degree of HSCs, the higher levels of α-SMA and Col1A1 will be, or conversely, the lower levels of α-SMA and Col1A1 [36]. As a result, α-SMA and Col1A1 are commonly used as markers to assess HSCs activation, with α-SMA being the most commonly used marker.

**Fig. 3:**
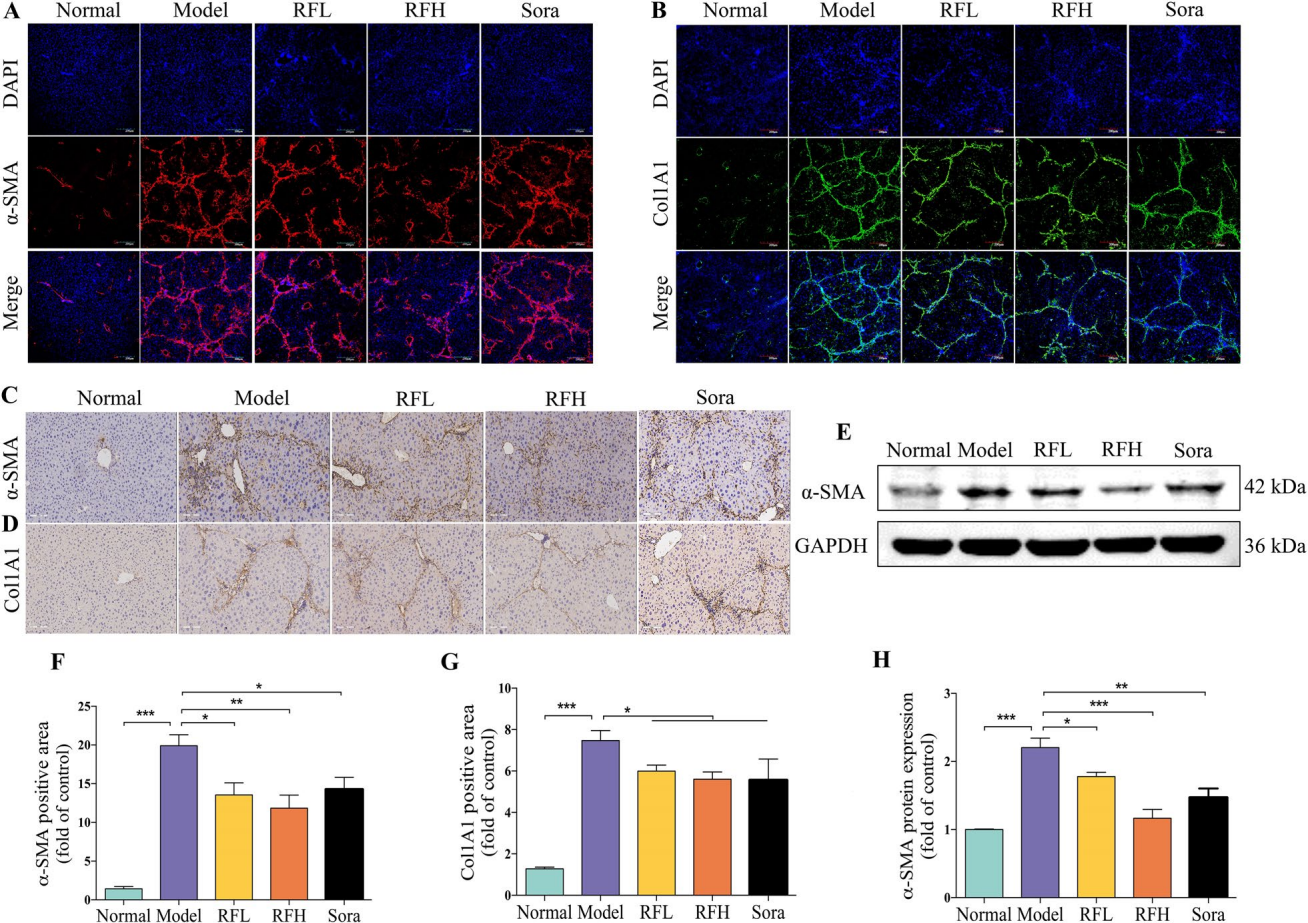
RF attenuates the expressions of α-SMA and Col1A1 in the liver of mice with CCl_4_-induced liver fibrosis. Representative immunofluorescence staining of **A** α-SMA and **B** Col1A1, the nuclei were counterstained with DAPI, representative confocal microscopy images are shown, scale bar = 200 μm. Representative immunohistochemical staining of **C** α-SMA and **D** Col1A1, scale bar = 100 μm. Quantification of histological changes of **F** α-SMA and **G** Col1A1 with positive area using Image J analysis software (n = 3). The expression of α-SMA was examined by **E** western blot assay and **H** its quantitative analysis (n = 3). The results are expressed as the mean ± SD. ^*^p < 0.05, ^**^ *p* < 0.01, ^***^ *p* < 0.001 versus the CCl_4_ group

Thus, in this study, α-SMA and Col1A1 were evaluated in HSCs activation. The expressions of α-SMA and Col1A1 on the protein levels were studied through immunohistochemistry, immunofluorescent and western blot assay. The expressions of α-SMA (Fig. 3A) and Col1A1 (Fig. 3B) were elevated greatly in the CCl_4_ group compared the control group in immunofluorescent assays. In contrast, the expressions of α-SMA and Col1A1 were suppressed in the RF and Sora treatment. Immunohistochemistry testing yielded identical results, as expected. In the normal group, α-SMA (Fig. 3C) and Col1A1 (Fig. 3D) were only slightly expressed around the blood vessels (the positive substance was brownish-yellow). Compared with the normal group, α-SMA (*p* < 0.001, Fig. 3F) and Col1A1 (*p* < 0.001, Fig. 3G) were significantly expressed in the fibrous septa and portal region in the model group. After RF and Sora treatment, in comparison to the model group, α-SMA (*p* < 0.05 for RFL; *p* < 0.01 for RFH; *p* < 0.05 for Sora) and Col1A1 (*p* < 0.05 for RFL, RFH and Sora) were significantly reduced. Western blot assay was employed to analyze the expression level of α-SMA protein (Fig. 3E). It was found that RF and Sora could cause a significant reduction in the expression level of α-SMA protein (*p* < 0.05 for RFL; *p* < 0.001 for RFH; *p* < 0.01 for Sora, Fig. 3H). In brief, the above results suggested that RF can ameliorate the activation of HSCs, which may be the potential mechanistic pathway regulating its liver fibrosis-mitigating impact in vivo.

### RF inhibits TGF-β/Smads signaling pathway

Activation of HSCs is regulated via various growth factors and inflammatory cytokines released from the impaired hepatocytes. Several cytokines are involved in the transformation and proliferation of HSCs, with TGF-β1 being the most effective [37, 38]. TGF-β1 is also involved in almost all stages of liver fibrosis. TGF-β1 can promote ECM synthesis, and inhibit the degradation of newly generated ECM, thereby disrupting ECM’s natural equilibrium and resulting in over-position of ECM and eventually exacerbated liver fibrosis. TGF-β1 promotes liver fibrosis formation through various mechanisms, among which the TGF-β/Smads signaling pathway has a key involvement in the progress of liver fibrosis by promoting HSCs transdifferentiation and migration [37]. TβRII receptor on the cell membrane binds to the activated TGF-β1, recognizing the TβRI receptor and phosphorylates at its glycine-serine enrichment region. Then, Smad2/3 protein is phosphorylated by the activated TβRI receptor and binds to Smad4 protein to form the Smads complex, which enters the nucleus. Smad4 enhances the activity of the Smad3 reaction promoter and promotes the formation and development of fibrosis by regulating the ability of Smad3 protein to transcriptome its target gene. After the Smads complex enters the nucleus, it binds to the target genes related to fibrosis. It regulates their transcription and expression to promote the activation of HSCs, thus promoting the occurrence of liver fibrosis [39–41]. As a result, inhibiting the TGF-β/Smads signaling pathway may be an effective strategy for inhibiting the activation of HSCs, thereby preventing the formation and progression of liver fibrosis.

**Fig. 4:**
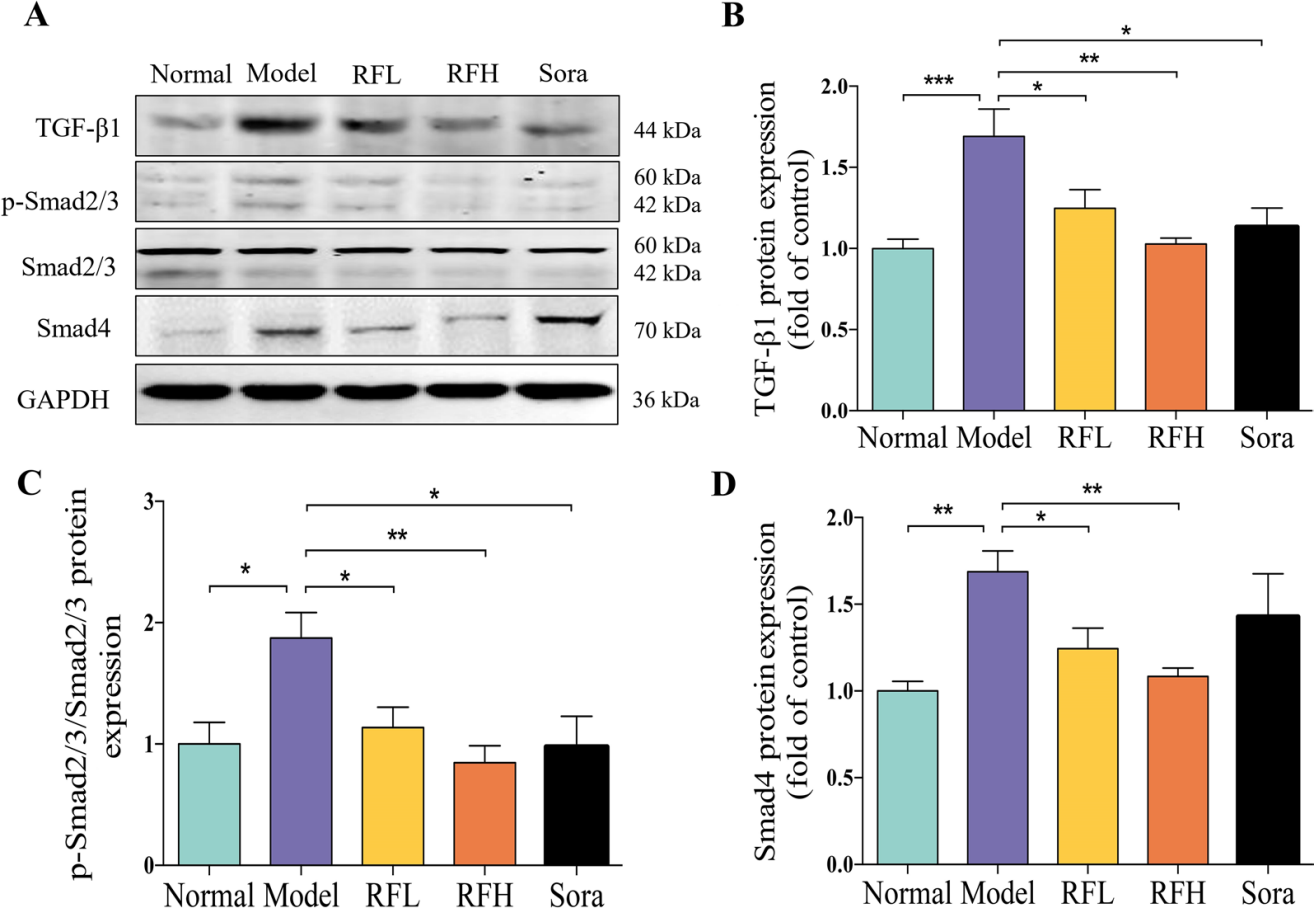
RF down-regulates TGF-β/Smads signaling pathway. The expression of **A** TGF-β1, p-Smad2/3, Smad2/3 and Smad4 were examined by western blot assay. Quantitative analysis of **B** TGF-β1, **C** p-Smad2/3/ Smad2/3, and **D** Smad4 expressions (n = 3). The results are expressed as the mean ± SD. ^*^p < 0.05, ^**^p < 0.01, ^***^p < 0.001 versus the CCl_4_ group

Our findings were confirmed by western blot assays of TGF-β/Smads signaling pathway-related proteins, including TGF-β1, Smad2/3, p-Smad2/3, and Smad4 (Fig. 4A). The protein expressions of TGF-β1 (*p* < 0.01, Fig. 4B), p-Smad2/3/Smad2/3 (*p* < 0.05, Fig. 4C), and Smad4 (*p* < 0.01, Fig. 4D) in the CCl_4_-induced fibrosis group were significantly higher than those in the control group. RF and Sora treatment clearly reduced the expression of TGF-β1 (*p* < 0.05 for RFL; *p* < 0.01 for RFH; *p* < 0.05 for Sora), p-Smad2/3/Smad2/3 (*p* < 0.05 for RFL; *p* < 0.01 for RFH; *p* < 0.05 for Sora) and Smad4 (*p* < 0.05 for RFL; *p* < 0.01 for RFH). The results suggested that RF inhibited TGF-β1 expression, and decreased the expressions of downstream signaling molecules p-Smad2/3 and Smad4 protein, which means the signal transduction ability of TGF-β1 was weakened, and then inhibited the activation and proliferation of HSCs, to play an anti-fibrosis role. The inhibitory effect of RF was concentration-dependent, and the higher the concentration of RF, the greater the inhibitory effect on the TGF-β/Smads signaling pathway, according to the results. The present findings revealed that RF strongly inhibits the TGF-β/Smads signaling pathway, which is linked to the relief of CCl_4_-induced hepatofibrogenesis in mice.

### RF ameliorates gut microbial dysbiosis in CCl_4_-induced hepatic fibrosis mice

With the advancement of research in the realm of gut microbiota, the precise link between gut microbiota and various diseases is becoming more apparent [10, 42, 43]. The liver is the organ that has the most direct interaction with the intestinal system and is therefore exposed to many bacterial components. The dysbiosis of the microbiome has been linked to a variety of liver diseases, and it may influence the degree of hepatic steatosis, inflammation, and fibrosis [11, 39, 44]. The CCl_4_-induced hepatic fibrosis model has been frequently utilized to assess the potential of natural compounds for liver protection due to its excellent reproducibility. In addition to leading to lipid peroxidation in liver cells, the gut microbiota has also been played a potentially important role in CCl_4_-induced liver fibrosis [45–47]. The community structure of the gut microbiota has been demonstrated to change when CCl_4_ is administered orally or intraperitoneally to induce hepatic fibrosis [45–48]. Dapito et al. found that the incidence of liver injury, fibrosis, and hepatocellular carcinoma induced by CCl_4_ was significantly lower in germ-free mice than in normal mice [49]. Mazagova et al. reported that the symbiotic microbiota might prevent hepatic fibrosis in germ-free mice induced by CCl_4_ [50], indicating that the gut microbiota is critical in developing CCl_4_-induced liver fibrosis and that regulating the gut microbiota may be beneficial in alleviating liver fibrosis. Therefore, we investigated the effect of RF on the structure and composition of gut microbiota to see if its protection against liver fibrosis is associated with the modulation of intestinal flora imbalance.

**Fig. 5:**
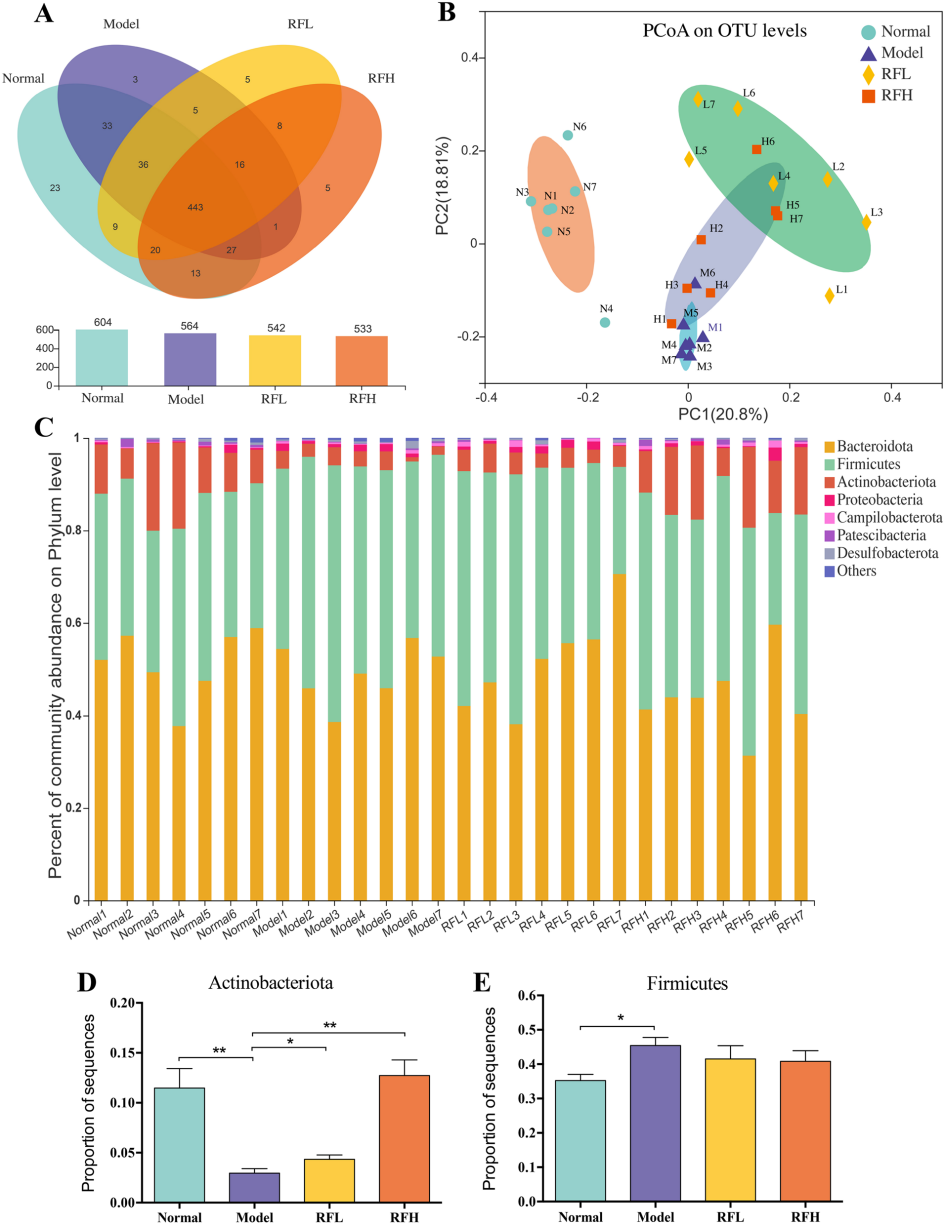
RF ameliorates gut microbial dysbiosis in CCl_4_-induced hepatic fibrosis mice. **A** Venn graph of the OTUs from gut microbiota of four groups. **B** Weighted UniFrac PCoA analysis of gut microbiota based on the OTU data of four groups. **C** The percent of community abundance on phylum-level in each mouse of four groups. Relative abundance of **D** Actinobacteriota and **E** Firmicutes in fecal microbiota in four groups. *p < 0.05, **p < 0.01 versus the CCl_4_ group

To examine the regulatory influence of RF on gut microbiota, we used the Illumina MiSeq technology to perform a pyrosequencing-based analysis of bacterial 16 S ribosomal RNA from variable regions V3–V4 of fecal samples. After removing the unqualified sequences, high-throughput pyrosequencing yielded an aggregate of 1,658,632 high-quality sequences from 28 fecal specimens (Additional file 1: Table S1). The Venn diagram highlighted how OTUs in the gut microbiota overlapped in different samples. A total of 647 OTUs were obtained from the samples using a 97% sequence similarity criterion, including 604 in the normal group, 564 in the model group, 542 in the RFL group, and 533 in the RFH group (Fig. 5A). 65 OTUs were found in the normal group but not in the model group, with 42 of these being found in the RFL and RFH groups. PCoA was used to estimate the level of similarity among gut microbiota compositions in four groups based on the weighted UniFrac distance of OTU abundance (Fig. 5B). There was a distinct grouping of gut microbial compositions for the normal and model groups. The RFL and RFH groups were grouped apart from the model group, indicating that RF caused a notable alteration in gut microbiota composition.

To evaluate the general structural framework of the bacterial community in various groups, we studied the extent of similarity of bacterial taxonomy at the phylum and genus levels. Firmicutes, Bacteroidetes, and Actinobacteriota were among the most prevalent phyla in the fecal microbiota community at the phylum level (Fig. 5C). The relative abundance of Actinobacteriota decreased (3.0 ± 1.2% vs. 11.5 ± 5.2%, *p* < 0.01, Fig. 5D) and that of Firmicutes increased (45.4 ± 6.1% vs. 35.2 ± 4.8%, *p* < 0.05, Fig. 5E) in the model group, in comparison to the normal group. Supplementation with RF did not significantly affect Firmicutes abundance (41.5 ± 10.1% vs. 45.4 ± 6.1% for RFL, *p* > 0.05; 40.8 ± 8.3% vs. 45.4 ± 6.1% for RFH, *p* > 0.05), but prevent the decrease of Actinobacteriota (4.3 ± 1.1% vs. 3.0 ± 1.2% for RFL, *p* < 0.05; 12.7 ± 4.1% vs. 3.0 ± 1.2% for RFH, *p* < 0.01) that was induced by the CCl_4_, suggesting that RF has the potential to carve the structure of gut microbiota.

**Fig. 6:**
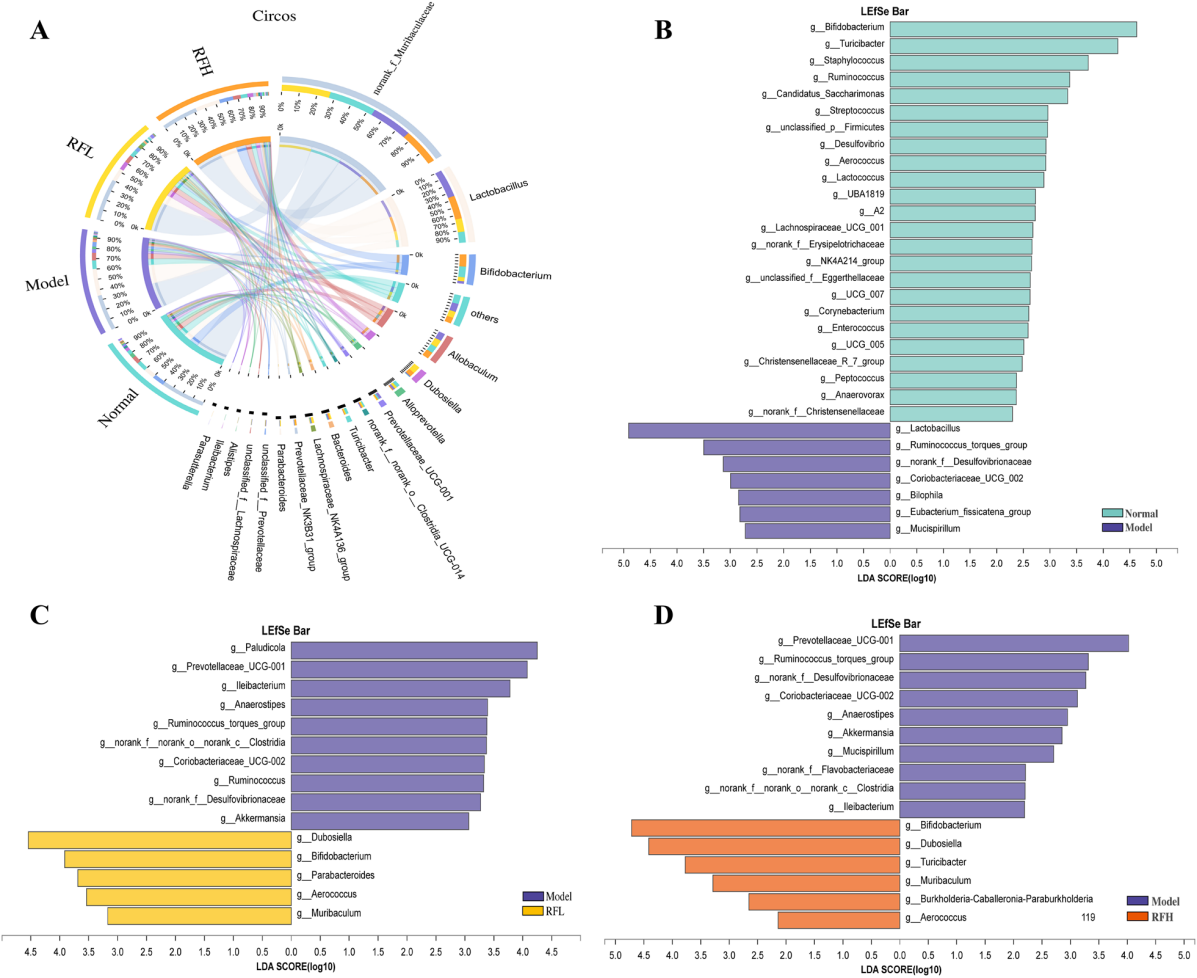
RF ameliorates gut microbial dysbiosis on genus level. **A** Circos diagram of microbial distributions on genus level for four groups. The LEfSe analysis of the gut microbiota differed between two groups (**B** Normal and Model groups; **C** Model and RFL groups; **D** Model and RFH groups). The statistical test was performed using LDA effect size method. The histogram showed the lineages with LDA values of 2.0 or higher as determined by LEfSe

At the genus level, 137 species of bacteria genera were discovered in the four sets of specimens, with 18 genera identified as the dominant genera with more than 1% relative abundance. Circos of dominating genera (Fig. 6A) and the LEfSe analysis further demonstrated that RF supplementation reversed the gut microbiota profile changes induced by CCl_4_. According to log10 LDA > 2.0, CCl_4_ had a substantial impact on 31 bacterial genera, with 24 genera having lower abundance and 7 genera having a higher abundance than the control group (Fig. 6B). Furthermore, this impact was clearly seen in three of the 31 bacterial taxa with log10 LDA > 4.0. Specifically, Bifidobacterium and Turicibacter were depleted remarkably upon exposure to CCl_4_, whereas Lactobacillus was enriched remarkably. A comparison between RFL and the model group showed that 5 genera (including Dubosiella, Bifidobacterium, Parabacteroides) were higher, and 10 genera were lower in the RFL group compared to the model group (Fig. 6C). For example, Dubosiella, Bifidobacterium, and Parabacteroides were elevated by RFL administration, alongside a decrease in Paludicola, Prevotellaceae_UCG-001, Ileibacterium. Further comparison between RFH and the model group showed that 6 genera were enriched and 10 genera were decreased in the RFH group than in the model group (Fig. 6D). For example, Bifidobacterium, Dubosiella, and Turicibacter were elevated by RFH administration, accompanied by a reduction in Prevotellaceae_UCG-001, Ruminococcus_torques_group, Norank_f_Desulfovibrionaceae. Collectively, a total of 18 different bacterial genera were increased or decreased by RF administration, and all these results indicated that RF administration restored the CCl_4_-induced imbalance of the gut microbiota to the levels of the normal group, RF at an elevated concentration manifested a more excellent capability in comparison the lower concentration.

### Correlation analysis between gut microbiota and liver fibrosis indexes

**Fig. 7:**
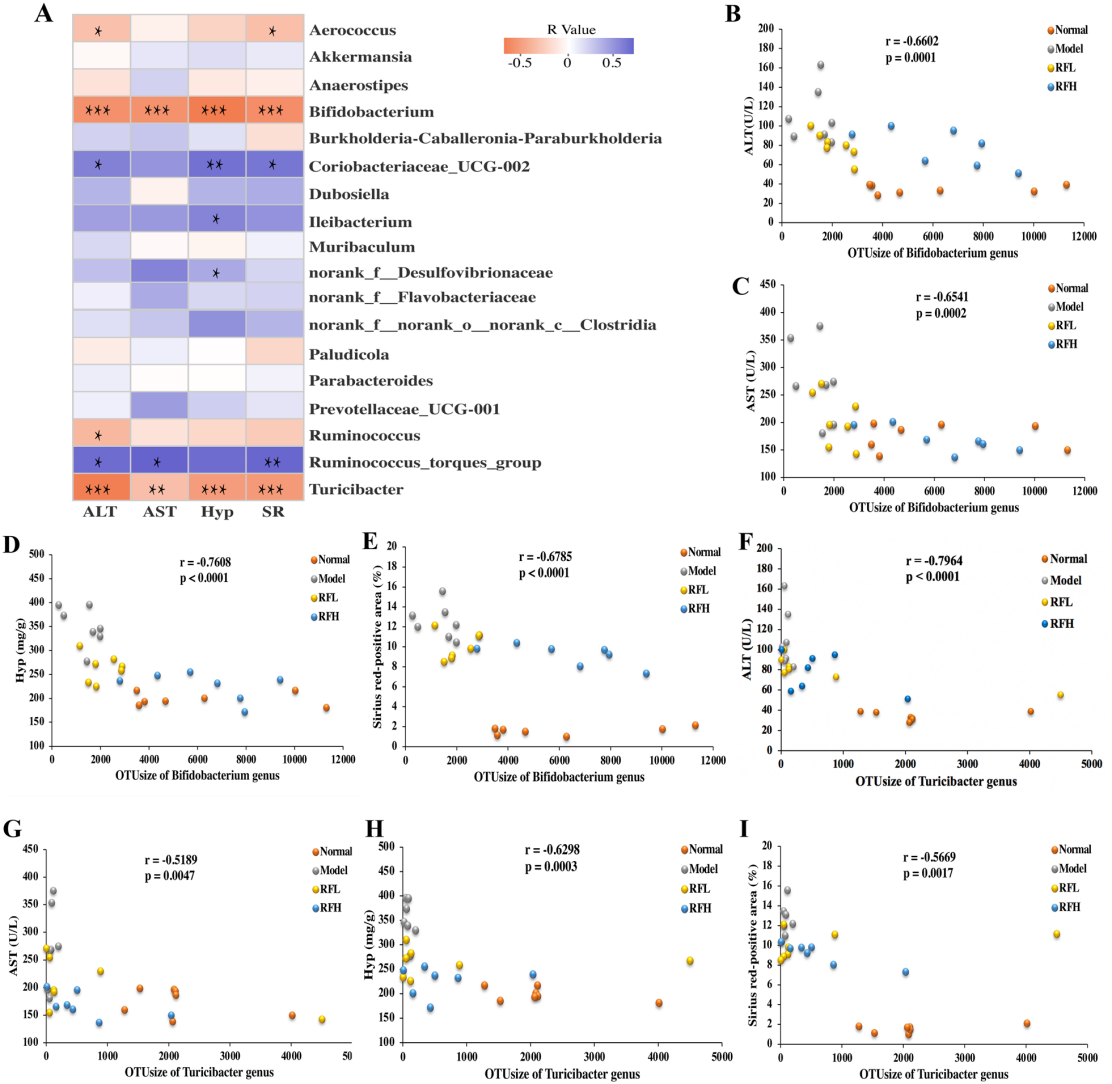
The correlation analysis of altered bacterial genera and pharmacodynamics parameters. **A** Spearman’s correlation between 18 altered bacterial genera by RF (Aerococcus, Akkermansia, Anaerostipes, Bifidobacterium, Burkholderia-Caballeronia-Paraburkholderia, Coriobacteriaceae UCG-002, Dubosiella, Ileibacterium, Muribaculum, norank_f_Desulfovibrionaceae, norank_f_Flavobacteriaceae, norank_f_norank_0_norank_c_Clostridia, Paludicola, Parabacteroides, Prevotellaceae_UCG-001, Ruminococcus, Ruminococcus_torques_group, Turicibacter) and 4 pharmacodynamics parameters (ALT, AST, Hyp, SR), the color scale represents the spearman r value, with blue and orange indicating positive and negative correlations, respectively, and *p < 0.05, ** *p* < 0.01, *** *p* < 0.001. Correlations of Bifidobacterium abundance with **B** ALT, **C** AST, **D** Hyp and **E** SR. Correlations of Turicibacter abundance with **F** ALT, **G** AST, **H** Hyp and **I** SR

Liver fibrosis has predisposed to gut microbiome dysfunction in animal models and patients [47, 51]. By promoting intrahepatic inflammatory responses and activating Toll-like receptor (TLR) related pathways, dysfunctional gut microbiota can aggravate liver fibrosis [52, 53]. Alleviating the gut microbiota disorder in liver fibrosis will improve liver inflammation and fibrosis, indicating the potential of regulating gut microbiota as a therapeutic or diagnostic method for liver fibrosis [51]. Our findings are consistent with those of other research in which the gut microbiota of CCl_4_-induced liver fibrosis mice was reported to be disrupted [45–47], and the supplementation of RF can effectively ameliorate liver fibrosis induced by CCl_4_ while modulating the gut microbiota simultaneously. To determine the interrelation between the gut microbiota-regulating effects of RF and its advantageous impact on liver fibrosis, we performed a spearman’s rank correlation analysis on 18 altered bacterial genera (Aerococcus, Akkermansia, Anaerostipes, Bifidobacterium, Burkholderia-Caballeronia-Paraburkholderia, Coriobacteriaceae_UCG-002, Dubosiella, Ileibacterium, Muribaculum, norank_f_Desulfovibrionaceae, norank_f_Flavobacteriaceae, norank_f_norank_0_norank_c_Clostridia, Paludicola, Parabacteroides, Prevotellaceae_UCG-001, Ruminococcus, Ruminococcus_torques_group, Turicibacter) through the RF as well as 4 pharmacodynamics parameters (ALT, AST, Hyp, SR) to determine whether there was a relationship. According to the findings, 8 bacterial genera were found to be significantly correlated with at least one pharmacodynamics parameter (*p* < 0.05). A total of 19 cases were found to be significantly associated (*p* < 0.05, Fig. 7A), of which Bifidobacterium (for ALT, r = − 0.66, *p* = 0.0001, Fig. 7B; for AST, r = − 0.65, *p* = 0.0002, Fig. 7C; for Hyp, r = − 0.76, *p* < 0.0001, Fig. 7D; for SR, r = − 0.68, *p* < 0.0001, Fig. 7E), and Turicibacter (for ALT, r = − 0.80, *p* < 0.0001, Fig. 7F; for AST, r = − 0.52, *p* = 0.0047, Fig. 7G; for Hyp, r = − 0.63, *p* = 0.0003, Fig. 7H; for SR, r = − 0.57, *p* = 0.0017, Fig. 7I) were found to be the two most pertinent bacterial genera. It indicates that Bifidobacterium and Turicibacter are linked to hepatic fibrosis severity. By altering the composition of the gut microbial population and partially boosting Bifidobacterium and Turicibacter, RF alleviated CCl_4_-induced hepatic fibrosis.

**Fig. 8:**
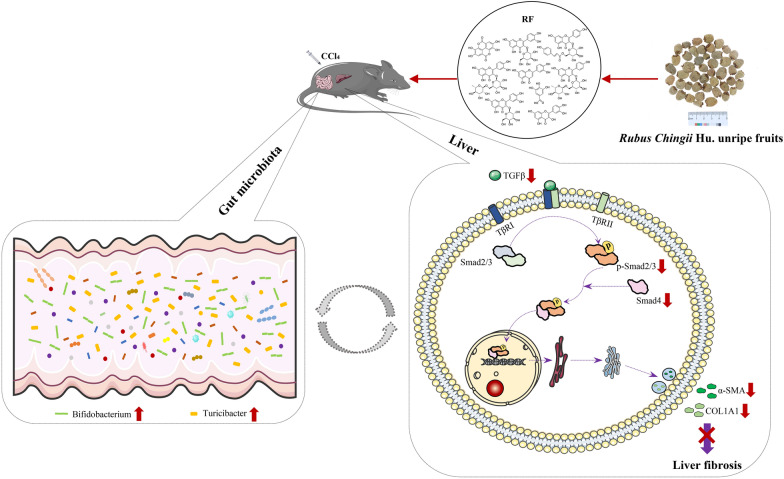
Proposed mechanism of RF in ameliorating CCl_4_-induced liver fibrosis. The red up arrow (↑) and red down arrow (↓) represent up-regulating and down-regulation effects of RF, respectively

According to the current study results, the higher Bifidobacterium and Turicibacter populations may be the two bacteria predominantly associated with RF’s anti-fibrosis effects on the liver. Bifidobacterium has been found as a probiotic that is practically ubiquitous in humans and is capable of producing anti-inflammatory short-chain fatty acids (SCFAs) [54]. Supplementing with Bifidobacterium has been shown to help alleviate liver steatosis, steatohepatitis, and fibrosis [55, 56]. Except for a few research that discovered changes in their abundance during liver fibrosis, there have been few investigations that have revealed Turicibacter’s specific role in liver fibrosis. Even though the precise functions of Bifidobacterium and Turicibacter, as well as their mechanism of action on liver fibrosis, are unknown, the available research suggests that raising Bifidobacterium and Turicibacter abundance can help with hepatic fibrosis recovery. Ursolic acid was found to prevent liver fibrosis in CCl_4_-induced liver fibrosis mice by suppressing HSC activation, correcting the gut microbiota imbalance, and increasing Bifidobacterium abundance [57]. Zhang et al. reported that mice with CCl_4_-induced acute liver injury had fewer Turicibacter, intake of an active substance-goats’ milk could protect against acute CCl4-induced hepatic injury, as evidenced by lower ALT and AST levels in serum, while also improving the gut microbiota imbalance, including an increase in Turicibacter abundance [48]. Our findings supported the notion that RF’s ability to regulate the gut microbial community, in part by increasing Bifidobacterium and Turicibacter, is associated with its anti-liver fibrosis effects. However, the molecular mechanism of RF affecting gut microbiota and its interaction with hepatic fibrosis needs further exploration. The current work demonstrates that RF has the potential to be developed as a novel prospective active agent for the treatment of liver fibrosis, and it offers a non-toxic and highly effective biological strategy for enriching the beneficial Bifidobacterium (Fig. 8).

In additional, oil-water partition coefficient (Log P) is an important material property of drugs. The dissolution, absorption, distribution, and transport of drugs in the body are related to their water solubility and lipid solubility. In present study, the Log P of target markers were obtained from literatures and professional databases (shown in Additional file 1: Table S3) [58–60], and the lipid solubility of these targeted markers were luteolin > tiliroside > kaempferol-3-rutinoside > quercetin > ellagic acid > isoquercitrin > hyperoside > rutin > gallic acid. As we all know, the lipid-soluble chemical components are more easily absorbed into the blood or into the body of intestinal bacteria through biological membranes. And then these components might be partially reversed the biochemical abnormalities and improved the associated gut microbiota imbalance. The water-soluble glycosides in the targeted markers might been have long retention time in the intestine, and these compounds could affect the composition of intestinal bacteria, and might be deglycosylated metabolism to lipid solubility aglycones by the intestinal bacteria. The complex interaction between the chemical components of traditional Chinese medicine and intestinal bacteria might be a difficulty in clarifying the pharmacodynamic mechanism of traditional Chinese medicine, and it might also be a breakthrough in analyzing the mechanism of traditional Chinese medicine, which is worthy of further research and exploration.

## Conclusions

The unripe fruits of *Rubus chingii* Hu. are a good source of nutrients, but their effects on organisms suffering from acute hepatic damage caused by CCl_4_ were previously unknown. According to the present study findings, RF can reduce liver fibrosis by altering TGF-β/Smad signaling by decreasing protein expression of Smad2/3 phosphorylation and Smad4 formation, which results in down-regulation of Col1A1 and α-SMA transcriptions. Moreover, the anti-liver fibrosis action of RF was associated with the regulation of the gut microbial community imbalance, which was partially accomplished in the liver fibrosis model by increasing Bifidobacterium and Turicibacter. Hence, to produce anti-liver fibrosis drugs or health-care products supplemented with RF, further research into the relationship between RF, its targeted bacteria, and anti-liver fibrosis function, as well as the exact mechanism, is required.

## Supplementary Information

**Additional file 1: Table S1.** The information of 28 fecal specimens by 16S ribosomal RNA analysis. **Table S2.** Ingredient identification of the extract of Rubus chingii Hu unripe fruits (RF) byUHPLC-Q-Exactive Orbitrap HRMS. **Table S3.** The Log P of target compounds in RF. **Figure S1.** The total ion chromatograms (TICs) of the extract of Rubus chingii Hu unripe fruits (RF).**A** negative mode; **B** positive mode. **Figure S2.** The ^1^H-NMR/^13^C-NMR and MS spectra of gallic acid (**A**), isoquercitrin (**B**) and ellagic acid(**C**). **Figure S3.** The ^1^H-NMR and MS spectra of hyperoside (**A**), rutin (**B**) and quercetin (**C**). **Figure S4.** The 1H-NMR and MS spectra of kaempferol-3-rutinoside (**A**), luteolin (**B**) and tiliroside (**C**).

## Data Availability

All the data used to support the fundings of this study are available from the corresponding author upon reasonable request.

## Abbreviations

α-SMA: α-smooth muscle actin
ALT: Alanine aminotransferase
AST: Aspertate aminotransferase
CCl_4_: Carbon tetrachloride
Col1A1: Type I collagen
ECM: Extracellular matrix
H&E: Hematoxylin and eosin
Hyp: Hydroxyproline
HSCs: Hepatic stellate cells
IL-1β: Interleukin-1β
IL-6: Interleukin-6
LDA: Linear discriminant analysis
LEFSe: Linear discriminant analysis effect size
MCP-1: Monocytechemoattractantprotein-1
OTUs: Operational taxonomic units
PCoA: Principal coordinate analysis
QIIME: Quantitative insights into microbial ecology
RF: The extract of *Rubus chingii* Hu. unripe fruits
RFH: High-dose RF group
RFL: Low-dose RF group
SR: Sirius red-positive area
SRA: Sequence read archive
TCM: Traditional Chinese medicine
TNF-α: Tumor necrosis factor-α
UHPLC-Q-Exactive Orbitrap: Ultra-high-performance liquid chromatography-Q exactive hybrid quadrupole orbitrap
HRMS: High-resolution accurate mass spectrometric
