# Supplementary material for: Rubus chingii Hu. unripe fruits extract ameliorates carbon tetrachloride-induced liver fibrosis and improves the associated gut microbiota imbalance
Source: Chin Med. 2022 May 12;17:56. doi: 10.1186/s13020-022-00607-6 (PMC9097331; doi:10.1186/s13020-022-00607-6)
Supplement: Supplementary file 1 — Additional file 1: Table S1. The information of 28 fecal specimens by 16S ribosomal RNA analysis. Table S2. Ingredient identification of the extract of Rubus chingii Hu unripe fruits (RF) byUHPLC-Q-Exactive Orbitrap HRMS. Table S3. The Log P of target compounds in RF. Figure S1. The total ion chromatograms (TICs) of the extract of Rubus chingii Hu unripe fruits (RF).A negative mode; B positive mode. Figure S2. The 1H-NMR/13C-NMR and MS spectra of gallic acid (A), isoquercitrin (B) and ellagic acid(C). Figure S3. The 1H-NMR and MS spectra of hyperoside (A), rutin (B) and quercetin (C). Figure S4. The 1H-NMR and MS spectra of kaempferol-3-rutinoside (A), luteolin (B) and tiliroside (C). [file 13020_2022_607_MOESM1_ESM.pdf]

# Supplementary information

## *for*

## *Rubus chingii* Hu. unripe fruits extract ameliorates carbon tetrachloride-induced liver fibrosis and improves the associated gut microbiota imbalance

Jianjun Wu [a, 1], Dingqi Zhang [b,1], Bo Zhu [a], Siqi Wang [a], Yongbin Xu [c],

Congcong Zhang [c], Hailing Yang [b], Shunchun Wang [c], Ping Liu [b], Luping Qin [a,*], Wei Liu [b,*]

[a] *College of Pharmaceutical Sciences, Zhejiang Chinese Medical University, Hangzhou, Zhejiang 310053, China.*

[b] *Key Laboratory of Liver and Kidney Diseases (Ministry of Education), Institute of Liver Diseases, Shuguang Hospital Affiliated to Shanghai University of Traditional Chinese Medicine, 528 Zhangheng Road, Shanghai 201203, China*

[c] *Institute of Chinese Materia Medica, Shanghai University of Traditional Chinese Medicine; The MOE Key Laboratory for Standardization of Chinese Medicines and The SATCM Key Laboratory for New Resources and Quality Evaluation of Chinese Medicines, 1200 Cailun Rood, Shanghai 201203, China*

*Correspondence to: Professor Luping Qin and Wei Liu. School of Pharmacy, Zhejiang Chinese Medical University, Hangzhou, Zhejiang 310053, China. Tel: 086-0571-61768167, Fax: 086-0571-61768136, E-mail: lpqin@zcmu.edu.cn; Institute of Liver Diseases, Shuguang Hospital Affiliated to Shanghai University of Traditional Chinese Medicine, 528 Zhangheng Road, Shanghai 201203, China. Tel: 086-021-20256526, Fax: 086-021-20256521, E-mail: lwhzayl@shutcm.edu.cn.

[1] Jianjun Wu and Dingqi Zhang contributed equally to this work.

## 1. RF ameliorates gut microbial dysbiosis in CCl4-induced hepatic fibrosis mice

The Illumina MiSeq technology was used to perform a pyrosequencing-based analysis of bacterial 16S ribosomal RNA from variable regions V3–V4 of fecal samples. After removing the unqualified sequences, high-throughput pyrosequencing yielded an aggregate of 1658632 high-quality sequences from 28 fecal specimens (Table S1).

**Table S1 The information of 28 fecal specimens by 16S ribosomal RNA analysis**

| Sample\Info | Seq_num | Base_num | Mean_length | Min_length | Max_length |
|---|---|---|---|---|---|
| Normal1 | 68849 | 29005748 | 421.2951 | 244 | 511 |
| Normal2 | 64450 | 27205781 | 422.1223 | 273 | 496 |
| Normal3 | 63366 | 26806377 | 423.0404 | 254 | 451 |
| Normal4 | 56640 | 24063507 | 424.8501 | 219 | 479 |
| Normal5 | 52449 | 22218728 | 423.6254 | 277 | 530 |
| Normal6 | 58353 | 24701016 | 423.3033 | 201 | 485 |
| Normal7 | 66736 | 28192696 | 422.4511 | 266 | 474 |
| Model1 | 66263 | 28117903 | 424.3379 | 252 | 511 |
| Model2 | 69883 | 29666588 | 424.518 | 203 | 444 |
| Model3 | 67485 | 28482030 | 422.0498 | 216 | 491 |
| Model4 | 68497 | 29022078 | 423.6985 | 218 | 527 |
| Model5 | 49971 | 21203210 | 424.3103 | 205 | 465 |
| Model6 | 59203 | 24861239 | 419.9321 | 277 | 449 |
| Model7 | 55308 | 23420256 | 423.4515 | 255 | 499 |
| RFL1 | 45198 | 19168032 | 424.0903 | 276 | 505 |
| RFL2 | 58171 | 24672185 | 424.132 | 236 | 441 |
| RFL3 | 46054 | 19578222 | 425.1145 | 252 | 434 |
| RFL4 | 58305 | 24354717 | 417.7123 | 255 | 491 |
| RFL5 | 71814 | 30475739 | 424.3704 | 278 | 472 |
| RFL6 | 46111 | 19608557 | 425.2468 | 276 | 524 |
| RFL7 | 72503 | 30667715 | 422.9855 | 254 | 477 |
| RFH1 | 53542 | 22721612 | 424.3699 | 335 | 509 |
| RFH2 | 61211 | 25928963 | 423.5997 | 249 | 450 |
| RFH3 | 52870 | 22439065 | 424.4196 | 277 | 432 |
| RFH4 | 53177 | 22558118 | 424.2082 | 267 | 432 |
| RFH5 | 58227 | 24750540 | 425.0698 | 204 | 432 |
| RFH6 | 59609 | 25199659 | 422.7492 | 267 | 432 |
| RFH7 | 54387 | 23041038 | 423.6497 | 253 | 474 |
| Total | 1658632 | | | | |

## 2. Chemical profiling of Rubus chingii Hu unripe fruits extract (RF) by UHPLC-Q-Exactive Orbitrap HRMS

Chromatographic separation was performed on an UHPLC-Q-Exactive Orbitrap system (Thermo

Fisher Scientific Inc., Grand Island, NY, USA). The UHPLC system consisted of a Thermo Scientific Dionex Ultimate 3000 Series RS pump coupled with a Thermo Scientific Dionex Ultimate 3000 Series TCC-3000RS column compartments and WPS-3000 autosampler controlled by Chromeleon 7.2 Software. The cooling autosampler was set at 4 °C and protected from light, and the column heater was set at 40 °C. A Waters ACQUITY UPLC BEH $C_{18}$ column (2.1 × 100 mm, 1.7 μm) was employed with the temperature set at 40 °C. The mobile phase consisted of A (methanol) and B (0.1% formic acid) at a flow rate of 0.3 mL•min$^{-1}$ and eluted with gradient elution: 0-4 min (4% A), 4-10 min (4%-12% A), 10-30 min (12%-70% A), 30-35 min (70% A), 35-38 min (70-95% A), 38-42 min (95% A), 42-45 min (4% A). The injection volume was 2 μL.

The mass spectrometer Q-Exactive Orbitrap system was connected to the UHPLC system *via* heated electrospray ionization and controlled by Xcalibur 4.1 software that was used for data collection and analysis. The electrospray ionization source was operated and optimized in negative and positive ionization mode. The optimized parameters of mass spectrometry were: capillary temperature: 325°C; sheath gas ($N_2$) flow rate: 45 arbitrary units; auxiliary gas ($N_2$) flow rate: 8 arbitrary units; sweep gas flow rate: 0 arbitrary units; spray voltage: 2.5 kV (negative), 3.5 kV (positive); S-lens RF level: 50V; auxilliary gas heater temperature, 300°C; scan mode: Full MS/dd-MS2 mode, which includes 1 first-level full scan (resolution 70000 FWHM) and 1 data-dependent secondary scan (resolution 17500 FWHM) 2 events, the scanning range is 80-1200 m/z, and the collision energy gradient is 20V, 50V, 100V.

A total of 89 chemicals were quickly and accurately identified from RF (Fig. S1), *via* comparison with the retention times and MS/MS spectra of the reference standards, reference literatures, Chemical Book and other databases. Table S2 listed the detailed information of the identified chemical constituents in RF.

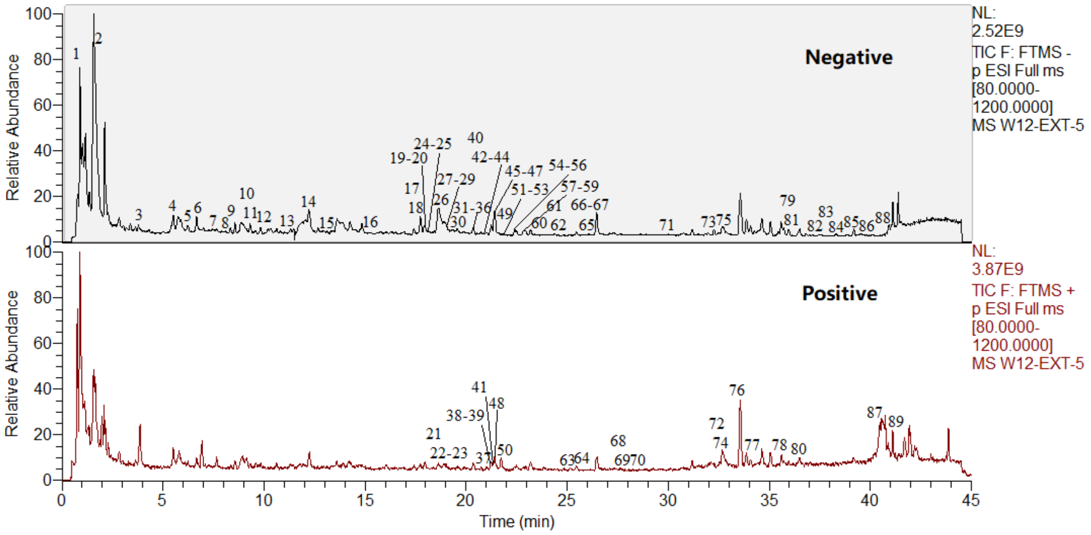


**Fig. S1 The total ion chromatograms (TICs) of the extract of *Rubus chingii* Hu unripe fruits (RF). A, negative mode; B, positive mode**

**Table S2 Ingredient identification of the extract of *Rubus chingii* Hu unripe fruits (RF) by UHPLC-Q-Exactive Orbitrap HRMS**

| NO. | tR/min | Ion model | Measured mass (Da) | Calculated mass (Da) | ppm | Formula | Identification |
|---|---|---|---|---|---|---|---|
| 1 | 0.93 | [M-H]$^-$ | 191.05518 | 191.05501 | 0.866 | $C_7H_{12}O_6$ | Quinic acid |
| 2* | 2.02 | [M-H]$^-$ | 169.01331 | 169.01315 | 0.948 | $C_7H_6O_5$ | Gallic acid |
| 3 | 4.12 | [M-H]$^-$ | 153.01813 | 153.01824 | -0.687 | $C_7 H_6 O_4$ | Protocatechuic acid |
| 4 | 5.74 | [M-H]$^-$ | 167.03391 | 167.03389 | 0.148 | $C_8H_8O_4$ | Vanillic acid |
| 5 | 6.84 | [M-H]- | 181.04982 | 181.04954 | 1.573 | $C_9H_{10}O_4$ | Syringaldehyde |
| 6 | 7.01 | [M-H]$^-$ | 577.13574 | 577.13405 | 2.924 | $C_{30}H_{26}O_{12}$ | Procyanidin B1 |
| 7 | 7.70 | [M-H]$^-$ | 577.13641 | 577.13405 | 4.085 | $C_{30}H_{26}O_{12}$ | Procyanidin B3 |
| 8 | 8.38 | [M-H]$^-$ | 289.07193 | 289.07066 | 4.377 | $C_{15}H_{14}O_6$ | Catechin |
| 9 | 8.67 | [M-H]$^-$ | 577.13550 | 577.13405 | 2.508 | $C_{30}H_{26}O_{12}$ | Procyanidin B2 |
| 10 | 9.53 | [M-H]$^-$ | 179.03400 | 179.03389 | 0.641 | $C_9H_8O_4$ | Caffeic acid |
| 11 | 9.61 | [M-H]$^-$ | 353.08807 | 353.08671 | 3.856 | $C_{16}H_{18}O_9$ | Neochlorogenic Acid |
| 12 | 10.11 | [M-H]$^-$ | 577.13574 | 577.13405 | 2.924 | $C_{30}H_{26}O_{12}$ | Procyanidin B4 |
| 13 | 11.86 | [M-H]$^-$ | 289.07190 | 289.07066 | 4.274 | $C_{15}H_{14}O_6$ | Epicatechin |
| 14 | 12.92 | [M-H]- | 163.03911 | 163.03897 | 0.139 | $C_9H_8O_3$ | p-Coumaric acid |
| 15 | 14.61 | [M-H]$^-$ | 353.08804 | 353.08670 | 3.771 | $C_{16}H_{18}O_9$ | Chlorogenic Acid |
| 16 | 15.52 | [M-H]$^-$ | 137.02321 | 137.02332 | -0.111 | $C_7H_6O_3$ | 3,4-Dihydroxybenzaldehyde |
| 17 | 18.15 | [M-H]$^-$ | 353.08820 | 353.08670 | 4.224 | $C_{16}H_{18}O_9$ | Cryptochlorogenic Acid |
| 18* | 18.19 | [M-H]$^-$ | 463.08875 | 463.08710 | 3.558 | $C_{21}H_{20}O_{12}$ | Isoquercitrin |
| 19* | 18.55 | [M-H]$^-$ | 300.99893 | 300.99789 | 3.443 | $C_{14}H_6O_8$ | Ellagic acid |
| 20* | 18.69 | [M-H]$^-$ | 463.08868 | 463.0871 | 3.407 | $C_{21}H_{20}O_{12}$ | Hyperoside |
| 21 | 18.70 | [M+H]$^+$ | 303.04953 | 303.04993 | -1.317 | $C_{15}H_{10}O_7$ | Herbacetin |
| 22 | 18.85 | [M+H]$^+$ | 465.10251 | 465.10275 | -0.521 | $C_{21}H_{20}O_{12}$ | Quercetin-4-O-B-D-Glucoside ,Herbacetin-7-O-B-D-Glucosi |

| | | | | | | | |
|---|---|---|---|---|---|---|---|
| | | | | | | | de |
| 23 | 18.93 | [M+H]+ | 611.15979 | 611.16066 | -1.425 | $C_{27}H_{30}O_{16}$ | Lucenin-2 |
| 24 | 18.94 | $[M-H]^-$ | 433.11401 | 433.11292 | 2.09 | $C_{21}H_{22}O_{10}$ | Chalconaringenin 4-O-Glucoside |
| 25* | 18.94 | $[M-H]^-$ | 609.14661 | 609.14501 | 2.625 | $C_{27}H_{30}O_{16}$ | Rutin |
| 26 | 19.00 | $[M-H]^-$ | 515.11963 | 515.11840 | 2.383 | $C_{25}H_{24}O_{12}$ | Isochlorogenic acid B |
| 27 | 19.41 | $[M-H]^-$ | 461.10928 | 461.10784 | 461.10784 | 461.10784 | Tectoridin |
| 28 | 19.53 | $[M-H]^-$ | 549.16174 | 549.16027 | 2.682 | $C_{26}H_{30}O_{13}$ | Naringenin 7-O-(2-B-D-Apiofuranosyl)-B-Dglucopyranoside |
| 29 | 19.61 | $[M-H]^-$ | 463.12518 | 463.12349 | 3.654 | $C_{22}H_{24}O_{11}$ | Hesperetin-O-Glucoside |
| 30 | 20.32 | $[M-H]^-$ | 447.09354 | 447.09219 | 3.024 | $C_{21}H_{20}O_{11}$ | Luteolin-7-O-B-D-Glucoside |
| 31 | 20.51 | $[M-H]^-$ | 431.09848 | 431.09727 | 2.799 | $C_{21}H_{20}O_{10}$ | Vitexin |
| 32 | 20.62 | $[M-H]^-$ | 515.11932 | 515.11840 | 1.781 | $C_{25}H_{24}O_{12}$ | Isochlorogenic acid A |
| 33 | 20.66 | $[M-H]^-$ | 547.14594 | 547.14461 | 2.418 | $C_{26}H_{28}O_{13}$ | Chrysin6-C-A-L-Arabinoside-8-C-B-D-Glucoside |
| 34 | 20.79 | $[M-H]^-$ | 267.06641 | 267.06519 | 4.586 | $C_{16}H_{12}O_4$ | Formononetin |
| 35 | 20.85 | $[M-H]^-$ | 609.18280 | 609.18140 | 2.304 | $C_{28}H_{34}O_{15}$ | Hesperidin |
| 36 | 20.87 | $[M-H]^-$ | 463.12497 | 463.12349 | 3.2 | $C_{22}H_{24}O_{11}$ | Hesperetin-O-Glucoside |
| 37 | 21.02 | $[M+H]^+$ | 195.06494 | 195.06519 | -1.258 | $C_{10}H_{10}O_4$ | Ferulic acid |
| 38 | 21.19 | $[M+H]^+$ | 287.05450 | 287.05501 | -1.729 | $C_{15}H_{10}O_6$ | Kaempferol |
| 39 | 21.19 | $[M+H]^+$ | 449.10770 | 449.10784 | -0.307 | $C_{21}H_{20}O_{11}$ | Kaempferol-3-O-Glucoside |
| 40 | 21.04 | $[M-H]^-$ | 447.09354 | 447.09219 | 3.024 | $C_{21}H_{20}O_{11}$ | Astragalin or isomer |
| 41 | 21.27 | $[M+H]^+$ | 463.12280 | 463.12349 | -1.485 | $C_{22}H_{22}O_{11}$ | Diosmetin-7-O-Glucoside |
| 42* | 21.04 | $[M-H]^-$ | 301.03543 | 301.03427 | 3.823 | $C_{15}H_{10}O_7$ | Quercetin |
| 43* | 21.21 | $[M-H]^-$ | 593.15167 | 593.15010 | 2.653 | $C_{27}H_{30}O_{15}$ | Kaempferol-3-Rutinoside |
| 44 | 21.42 | $[M-H]^-$ | 515.11938 | 515.11840 | 1.898 | $C_{25}H_{24}O_{12}$ | Isochlorogenic acid C |
| 45 | 21.55 | $[M-H]^-$ | 301.07181 | 301.07066 | 3.804 | $C_{16}H_{14}O_6$ | Hesperetin |
| 46 | 21.66 | $[M-H]^-$ | 651.15735 | 651.15558 | 2.725 | $C_{29}H_{32}O_{17}$ | 3',3,5-Hydroxy-4',5'-Methoxyflavone-O-Glucoside-orhamnoside |
| 47 | 21.73 | $[M-H]^-$ | 547.14587 | 547.14461 | 2.29 | $C_{26}H_{28}O_{13}$ | Chrysin 6-C-Pen-8-C-Hex |
| 48 | 21.74 | $[M+H]^+$ | 373.12769 | 373.12818 | -1.312 | $C_{20}H_{20}O_7$ | Sinensetin |
| 49 | 21.97 | $[M-H]^-$ | 623.16229 | 623.16066 | 2.614 | $C_{28}H_{32}O_{16}$ | Isorhamnetin-3-O-Rutinoside |
| 50 | 21.99 | $[M+H]^+$ | 625.17590 | 625.17631 | -0.659 | $C_{28}H_{32}O_{16}$ | Stellarin-2 |
| 51 | 22.09 | $[M-H]^-$ | 429.08270 | 429.08162 | 2.51 | $C_{21}H_{18}O_{10}$ | Chrysin7-O-B-D-Glucuronide |
| 52 | 22.23 | $[M-H]^-$ | 463.12531 | 463.12349 | 3.934 | $C_{22}H_{24}O_{11}$ | Hesperetin-O-Glucoside |
| 53 | 22.40 | $[M-H]^-$ | 563.14087 | 563.13953 | 2.376 | $C_{26}H_{28}O_{14}$ | Schaftoside |
| 54 | 22.66 | $[M-H]^-$ | 461.10941 | 461.10784 | 3.41 | $C_{22}H_{22}O_{11}$ | (2S)-5,7-Dihydroxy-6-Methoxyflavanone7-O-B-D-Glucuronide |
| 55 | 22.74 | $[M-H]^-$ | 489.10419 | 489.10275 | 2.939 | $C_{23}H_{22}O_{12}$ | 5,7-Dihydroxy-8,2'-Dimethox |

| | | | | | | | |
|---|---|---|---|---|---|---|---|
| | | | | | | | yflavone7-O-B -D-Glucuronide |
| 56 | 22.90 | [M+FA-H]$^-$ | 491.11957 | 491.11840 | 2.377 | $C_{22}H_{22}O_{10}$ | Tilianin |
| 57 | 23.13 | [M+FA-H]$^-$ | 491.11957 | 491.11840 | 2.377 | $C_{22}H_{22}O_{10}$ | Trifolirhizin |
| 58 | 23.15 | [M-H]$^-$ | 263.12885 | 263.12779 | 4.045 | $C_{15}H_{20}O_4$ | (+)-Abscisic acid |
| 59 | 23.15 | [M-H]$^-$ | 563.14093 | 563.13953 | 2.483 | $C_{26}H_{28}O_{14}$ | Isoschaftoside |
| 60 | 23.6 | [M-H]$^-$ | 431.09848 | 431.09727 | 2.799 | $C_{21}H_{20}O_{10}$ | Kaempferol-3-rhamnoside |
| 61 | 24.47 | [M-H]$^-$ | 577.15656 | 577.15518 | 2.388 | $C_{27}H_{32}O_{14}$ | Naringin |
| 62* | 24.99 | [M-H]$^-$ | 285.04071 | 285.03936 | 4.72 | $C_{15}H_{10}O_6$ | Luteolin |
| 63 | 25.15 | [M+H]+ | 579.17023 | 579.17083 | -1.039 | $C_{27}H_{30}O_{14}$ | Violanthin |
| 64 | 25.57 | [M+H]$^+$ | 361.09106 | 361.09179 | -2.032 | $C_{18}H_{16}O_8$ | Isoirigenin |
| 65 | 26.32 | [M-H]$^-$ | 313.07199 | 313.07066 | 4.234 | $C_{17}H_{14}O_6$ | 3’,4’-Dihydroxy-7,5’-Dime Thoxyflavone |
| 66* | 26.36 | [M-H]$^-$ | 593.13043 | 593.12897 | 2.466 | $C_{30}H_{26}O_{13}$ | Tiliroside |
| 67 | 26.62 | [M-H]$^-$ | 547.14563 | 547.14461 | 1.851 | $C_{26}H_{28}O_{13}$ | Chrysin 6-C-B -D-Glucoside- 8-C-A-L-Arabinoside |
| 68 | 27.24 | [M+H]$^+$ | 317.06519 | 317.06558 | -1.227 | $C_{16}H_{12}O_7$ | Irilin D |
| 69 | 27.59 | [M+H]$^+$ | 361.09199 | 361.09179 | -1.672 | $C_{18}H_{16}O_8$ | Irigenin |
| 70 | 28.53 | [M+H]+ | 301.07004 | 301.07066 | -2.075 | $C_{16}H_{12}O_6$ | Pratensein |
| 71 | 30.10 | [M+FA-H]$^-$ | 711.39502 | 711.39697 | 1.952 | $C_{36}H_{58}O_{11}$ | Nigaichigoside 1 |
| 72 | 32.59 | [M+Na]+ | 801.38792 | 801.38751 | -0.411 | $C_{37}H_{62}O_{17}$ | Goshonoside G |
| 73 | 32.68 | [M+FA-H]$^-$ | 691.35355 | 691.35516 | 1.613 | $C_{32}H_{54}O_{13}$ | 15,18-Di-*O*-β-D-glucopyranosyl-13(*E*)-*ent*-labda-7(8),13(14)- diene-3β,15,18–triol |
| 74 | 32.79 | [M+Na]+ | 801.38792 | 801.38751 | -0.411 | $C_{37}H_{62}O_{17}$ | 15-*O*-β-D-apiofuranosyl-(1→2)β-D-glucopranosyl-18-*O*-β-Dglucopyranosyl-13(E)-ent-labda-8(9),13(14)-diene-3β,15,18-triol |
| 75 | 33.46 | [M+FA-H]$^-$ | 691.35355 | 691.35516 | 1.613 | $C_{32}H_{54}O_{13}$ | 15,18-Di-*O*-β-D-glucopyranosyl-13(*E*)-*ent*-labda-8(9),13(14)-diene-3β,15,18–triol |
| 76 | 33.71 | [M+H]$^+$ | 403.13815 | 403.13874 | -1.474 | $C_{21}H_{22}O_8$ | Nobiletin |
| 77 | 34.58 | [M+Na]+ | 639.33510 | 639.33478 | -0.318 | $C_{31}H_{52}O_{12}$ | Goshonoside F6 |
| 78 | 35.69 | [M+H]$^+$ | 373.12840 | 373.12818 | -1.58 | $C_{20}H_{20}O_7$ | Tangeretin |
| 79 | 36.33 | [M-H]$^-$ | 503.33672 | 503.33820 | 1.484 | $C_{30}H_{48}O_6$ | Sericic acid |
| 80 | 36.39 | [M+H]$^+$ | 485.32615 | 485.32596 | -0.191 | $C_{30}H_{44}O_5$ | Fupenzic acid |
| 81 | 36.42 | [M-H]$^-$ | 501.32107 | 501.32239 | 1.324 | $C_{30}H_{46}O_6$ | 2α,19α,24-trihydroxyurs-12-ene-3-oxo-28-acid |
| 82 | 37.32 | [M-H]$^-$ | 503.33672 | 503.33820 | 1.484 | $C_{30}H_{48}O_6$ | Hyptatic acid |

| | | | | | | | |
|---|---|---|---|---|---|---|---|
| 83 | 38.21 | [M+FA-H]$^-$ | 675.35863 | 675.36047 | 1.837 | $C_{32}H_{54}O_{12}$ | Goshonoside F7 |
| 84 | 38.74 | [M-H]$^-$ | 487.34180 | 487.34299 | 1.189 | $C_{30}H_{48}O_5$ | Arjunolic acid |
| 85 | 39.42 | [M-H]$^-$ | 487.34180 | 487.34299 | 1.189 | $C_{30}H_{48}O_5$ | Euscaphic acid |
| 86 | 40.41 | [M-H]$^-$ | 485.32615 | 485.32751 | 1.359 | $C_{30}H_{46}O_5$ | 2α,19α-dihydroxy-3-oxo-12-ursen-28-oic acid |
| 87 | 40.57 | [M+H]$^+$ | 337.23733 | 337.23447 | -2.866 | $C_{20}H_{32}O_4$ | *ent*-16α,17-dihydroxy-kauran-19-oic acid |
| 88 | 41.28 | [M-H]$^-$ | 487.34180 | 487.34299 | 1.189 | $C_{30}H_{48}O_5$ | Tormentic acid |
| 89 | 41.52 | [M+Na]+ | 495.34448 | 495.34399 | -0.491 | $C_{30}H_{48}O_4$ | Maslinic acid |

* means that the ingredient was confirmed by the reference substance.

## 3. The NMR and MS spectra for targeted markers of RF

The $^1$H-NMR and MS spectra for isoquercitrind, hyperoside, rutin, quercetin, kaempferol-3-rutinoside, luteolin and tiliroside in RF were showed in Fig. S2-Fig. S4. The $^{13}$C-NMR and MS spectra for gallic acid and ellagic acid were showed in Fig. S2.

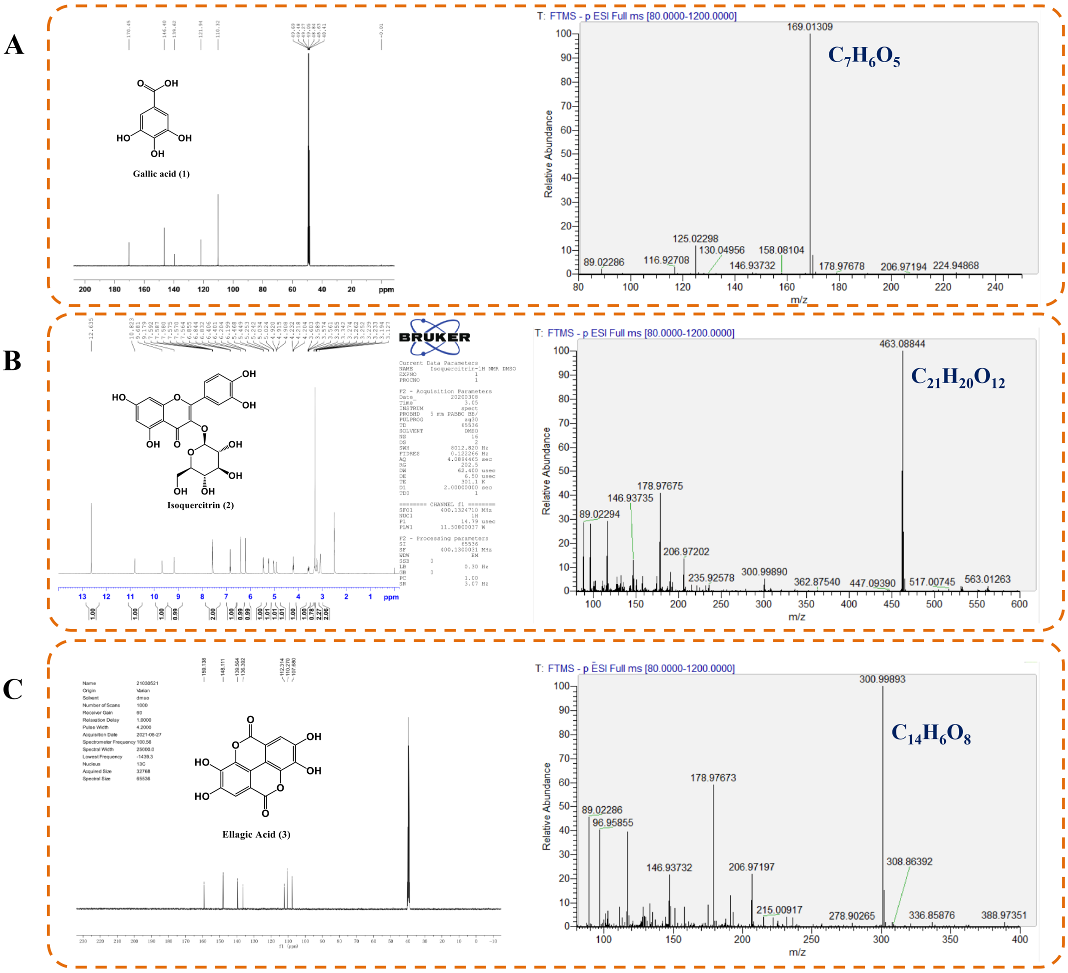


Fig. S2 The $^1$H-NMR/$^{13}$C-NMR and MS spectra of gallic acid (A), isoquercitrin (B) and ellagic acid (C)

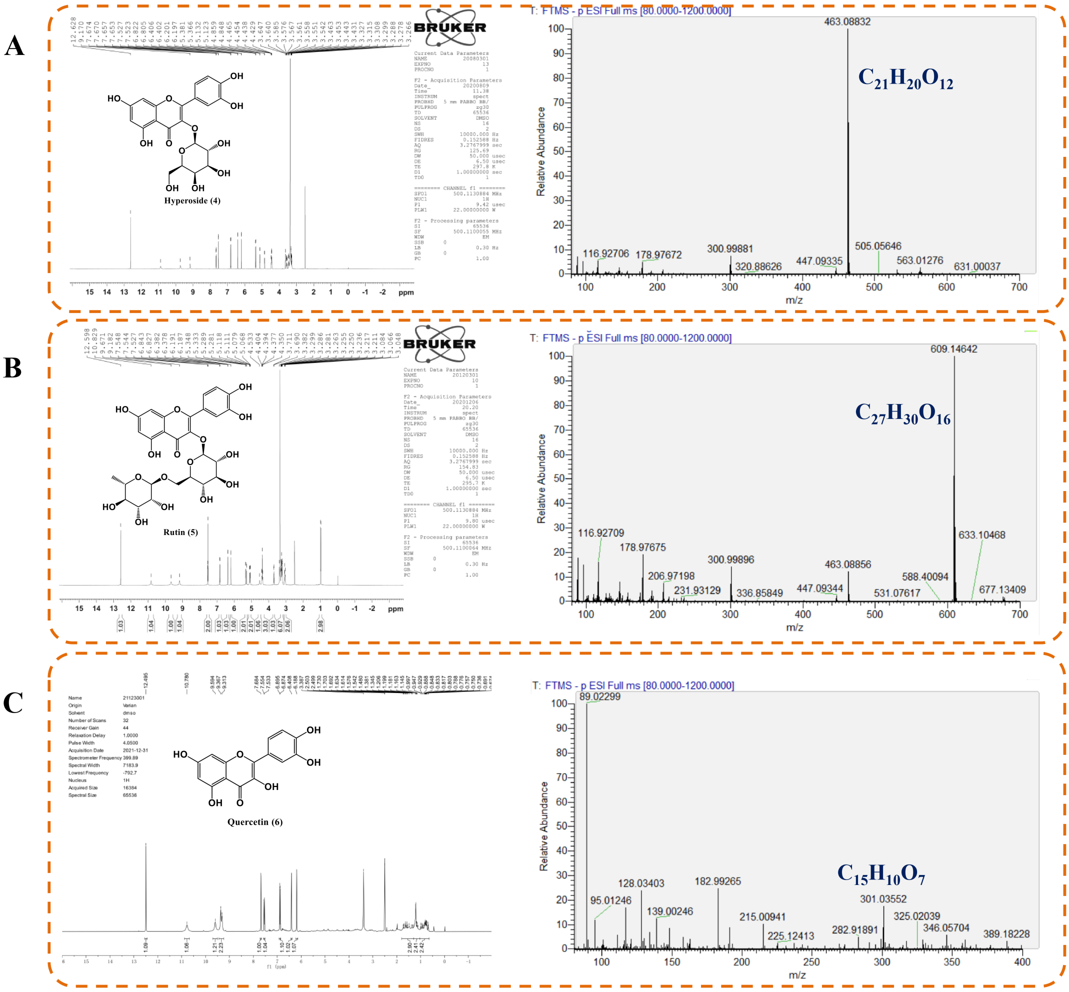


Fig. S3 The $^{1}$H-NMR and MS spectra of hyperoside (A), rutin (B) and quercetin(C)

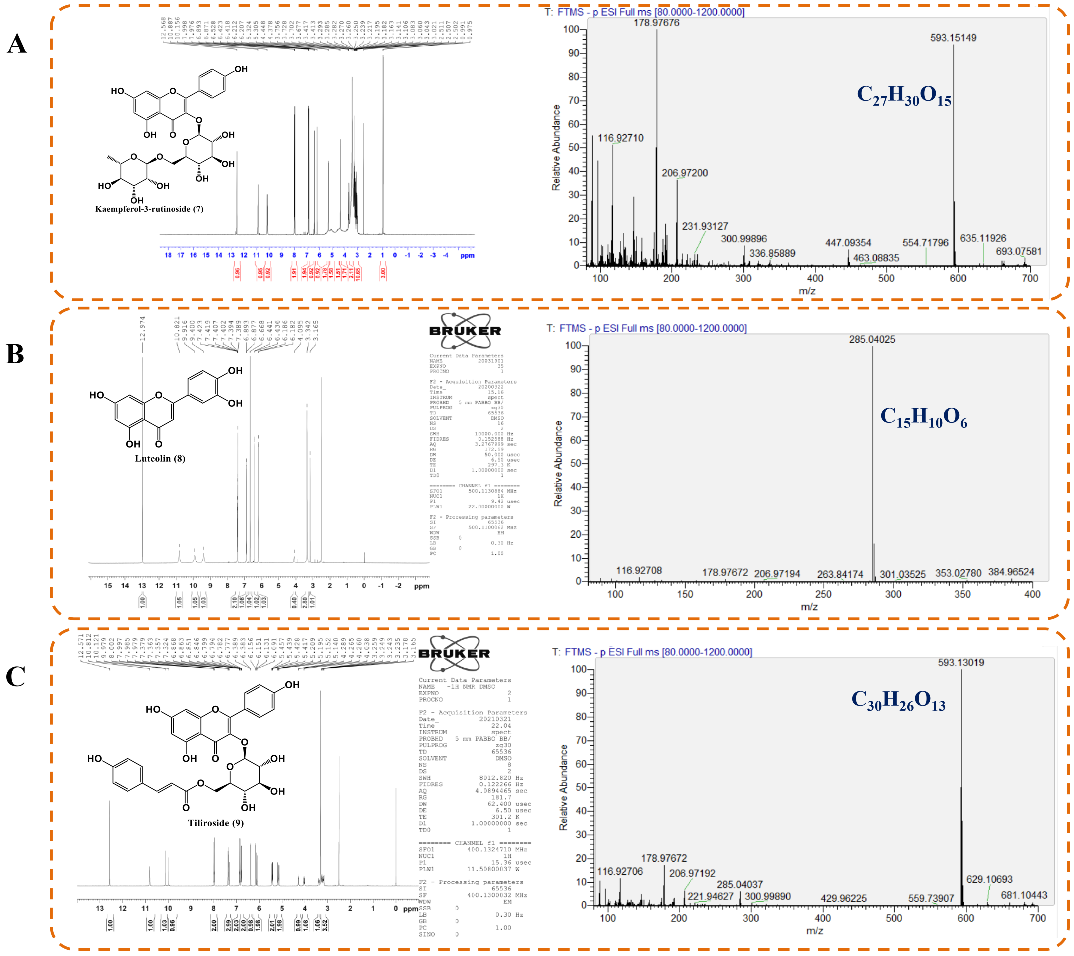


Fig. S4 The $^{1}$H-NMR and MS spectra of kaempferol-3-rutinoside (A), luteolin (B) and tiliroside (C)

## 4. The LogP of targeted markers of RF

The oil-water partition coefficient (Log P) of gallic acid, isoquercitrin, ellagic acid, hyperoside, rutin, kaempferol-3-rutinoside, quercetin, luteolin and tiliroside were obtained from literature and professional databases, and showed in Table S3.

**Table S3 The Log P of target compounds in RF**

| No. | Target compounds | LogP | Source |
|---|---|---|---|
| 1 | gallic acid | 0.7 | PubChem |
| 2 | isoquercitrin | 1.28 | Wu *et al.*, 2005 |
| 3 | ellagic acid | 1.59 | Truzzi *et al.*, 2021 |
| 4 | hyperoside | 1.26 | Wu *et al.*, 2005 |
| 5 | rutin | 1.18 | Wu *et al.*, 2005 |
| 6 | quercetin | 1.82 | Luo *et al.*, 2011 |
| 7 | kaempferol-3-rutinoside | 1.96 | Chemsrc |
| 8 | luteolin | 2.89 | Luo *et al.*, 2011 |
| 9 | tiliroside | 2.71 | Luo *et al.*, 2011 |

PubChem: https://pubchem.ncbi.nlm.nih.gov/; Chemsrc: https://www.chemsrc.com/en/
